# Supplementary material for: An energy-free strategy to elevate anti-icing performance of superhydrophobic materials through interfacial airflow manipulation
Source: Nat Commun. 2024 Jan 26;15:777. doi: 10.1038/s41467-024-45078-5 (PMC10817900; doi:10.1038/s41467-024-45078-5)
Supplement: Supplementary file 1 — Supplementary Information [file 41467_2024_45078_MOESM1_ESM.pdf]

SUPPLEMENTARY INFORMATION

An energy-free strategy to elevate anti-icing performance of superhydrophobic materials through interfacial airflow manipulation

Jiawei Jiang, Yizhou Shen, Yangjiangshan Xu, Zhen Wang, Jie Tao, Senyun Liu Weilan Liu, Haifeng Chen

1. Supplementary Discussion

1.1 Microstructure design based on drag reduction

1.1.1 Pressure Drag

Supplementary Figure 1 shows the variation of pressure around a microstructure unit with different angle. Pressure contour map illustrates that small areas with high pressure and low pressure appear on the windward side and slip face of the microstructure respectively when the angle is raised from 20° to 25°. Meanwhile, the difference between the high and the low-pressure areas is also augmented, indicating an increment of pressure gradient. As the angle is raised to 30°, the pressure difference between the front and rear of the microstructure still augments. When the angle is further reached to 35°, the negative pressure area on the slip face side is reduced significantly. Simultaneously, a region of high pressure over 50 Pa appears on the windward side, which improves the pressure difference significantly. Subsequently, as shown in Supplementary Fig. 1e, it is indicated that the area of high pressure on the windward side significantly expands to the entire microstructure as the angle is up to 40°, resulting in a higher pressure gradient. The continuous enlargement of the pressure difference at the front and rear of the microstructure brings a continuous improvement of pressure drag which is consistent with the result of the resistance analysis. Furthermore, as the angle rises, the number of structures in the same calculation region increases accordingly, resulting in a higher accumulation of pressure drag.

1.1.2 Viscous Resistance

Careful consideration is given to characterize the flow field with different angle in order to fully understand the variation of viscous resistance. The corresponding velocity streamlines diagram of angle is presented in Supplementary Fig. 2. It is obvious that the denseness of the streamlines in the micro-vortex remains steady, indicating a stable rotation velocity of micro-vortex with the growth of the angle. It is verified that a relatively stable velocity gradient is achieved between the micro-vortex and the upper fluid during the augment of the angle. In addition, the micro-vortex in the microstructure with an angle of 20° occupies about half of the microstructure in the flow direction, as displayed in Supplementary Fig. 2a. Nevertheless, when the angle of the microstructure is increased to 40°, the micro-vortex occupies approximately 80% of the microstructural area in the flow direction, as shown in Supplementary Fig. 2e. Notably, fluid will flow from the top of the microstructure units instead of along the inner wall of the microstructure units due to the presence of micro-vortex[1]. The gas-gas contact between the micro-vortex and external fluid replaces the original gas-solid contact between the fluid and the wall. The micro-vortex is similar to the roller bearing that converts sliding friction (caused by the wall and fluid) into rolling friction, leading to friction reduction, as shown in Supplementary Fig. 3.

Based on the “rolling bearing” effect, it is suggested that the size of the micro-vortex augments gradually in the flow direction with the augment of angle,

leading to a reduction of the contact area between the fluid and the microstructure. As mentioned above, the increscent size of micro-vortex can effectively reduce the positive viscous resistance. Moreover, it is worth noting that the shape of the micro-vortex is greatly affected by the microstructure. The bigger the angle of the microstructure, the easier it is to accumulate the low-speed fluid at the bottom of the microstructure, lifting the micro-vortex. Thereby, the distance between the micro-vortex and the microstructure surface extends with the growth of angle, which probably result in a decline of reverse velocity gradient near the surface.

1.1.3 Wall Shear Stress

The units of wall shear stresses with different angle are listed in Supplementary Fig. 4. It can be found that both the positive and the negative wall shear stress possess a same downward trend with the enlargement of angle. Moreover, the viscous resistance of a microstructure unit obtained by the integral of the wall shear stress curve has a declining tendency identically. According to the velocity distribution analysis above, the influence of the angle with tiny variation on boundary layer thickness should be slightly since the height of the microstructure is limited to 50 μm. Thus, it can be confirmed that the increased size of micro-vortex can effectively decrease the contact area between fluid and microstructure, leading to a reduction of positive viscous resistance. However, the removed micro-vortex dominates a continuous decreasing of reverse viscous resistance, resulting in a reduction of reverse

velocity gradient near the surface. The corresponding curve of reverse velocity gradient is shown in the Supplementary Fig. 5. It is also evidenced that the curve of reverse velocity gradient moves towards to right with the growth of angle, demonstrating a reduction of reverse velocity gradient.

1.2 Superposition of superhydrophobic property on microstructure

1.2.1 Movement Behavior of Droplets on Superhydrophobic Surface

In order to investigate the motion characteristics of droplets on superhydrophobic surfaces, the German Kruss K100 adhesion tester is adopted to measure the droplet adhesion during the directional movement process. In this work, the movement direction of the droplet facing the windward side of the microstructure is defined as direction 1, while the opposite direction is defined as direction 2 since the arrayed microstructures have directionality. Meanwhile, the directions along the ridge line of the microstructure are defined as directions 3 and 4, respectively, as shown in Supplementary Fig. 6a. The droplet used in this test is ultra-pure water with a volume of 20 μL. The droplet movement speed is recognized as 40 mm min^{-1}, and the movement distance is set to 18mm to ensure that the droplet always moves within the microstructure region. As a contrast, the dynamic adhesion of droplets in two vertical directions for the superhydrophobic plate with micro-nanostructures is also carried out to verify whether the superhydrophobic micro-nanostructures obtained by electrodeposition have isotropic wettability.

The results indicate that the adhesion forces of droplets moving in two

vertical directions on the superhydrophobic plate is 0.0186 N and 0.0193 N, respectively. It is clear that the superhydrophobic micro-nanostructures obtained by electrodeposition have isotropic wettability, as shown in Supplementary Fig. 6b. However, the adhesion force of the droplet moving along the direction 1 on A-20 sample is 0.0006 N, which is only 13% of that in the opposite direction, as shown in Supplementary Fig. 6c. This demonstrates that droplets are inclined to slide towards the windward side in the direction of microstructure arrangement. It is worth noting that the droplet adhesion forces are 0.0005 N and 0.00056 N in directions 3 and 4 respectively when droplets move along the ridge line of microstructures, which is little different from the droplet adhesion force in direction 1, as displayed in Supplementary Fig. 6d. This clarifies that the droplet may move randomly along ridge direction or towards the windward of the microstructures when sliding on the superhydrophobic A-20 surface.

Additionally, the adhesion force of the droplet moving towards the windward side on A-30 sample is 0.0012 N, which is 17.9% of that in the opposite direction, as illustrated in Supplementary Fig. 6e. Interestingly, the adhesive force of droplets rolling along the ridge line is between 0.0011 N and 0.0018 N, which is slightly higher than that moving towards the windward side, as demonstrated in Supplementary Fig. 6f. Hence, unlike the A-20 sample, it can be assumed that the droplet on A-30 sample is apt to sliding towards the windward side. Moreover, the adhesive force is measured to be 0.006 N when

the droplet moves towards the windward side on the A-40 sample, while the adhesive force of the droplet rolling in the opposite direction is 0.0099 N, as shown in Supplementary Fig. 6g. Simultaneously, the adhesive force of the droplets moving along the ridge line of the microstructure is only about 0.004 N, which means that the droplets tend to slide along the ridge line of microstructures on the A-40 sample.

Generally, the adhesive force of droplets moving towards the windward side is always smaller than that of droplets rolling in the reverse direction on various superhydrophobic surface with arrayed microstructures. Nevertheless, the adhesive force of droplets shifting along the ridge line of the microstructure is relatively similar. Notably, the difference in adhesion force of droplets moving in directions 1 and 2 gradually decreases and the adhesion force of droplets sliding along the ridge direction also gradually increases with the enlargement of the microstructure angle.

1.2.2 The Influence of Superhydrophobic Structure on the Drag

In order to explore the influence of superhydrophobic structure on the drag, the three treated samples were subjected to resistance analysis, as shown in Supplementary Fig. 7. It can be seen that the difference in drag reduction of these three samples is controlled within 10%, even if there are some aggregate microparticles on the surface. This indicates that superhydrophobic structure has little effect on aerodynamic performance of arrayed microstructure.

1.2.3 Surface Topography

The surface appearances of A-30 and A-40 samples are shown in Supplementary Fig. 8. It can be seen from the figure that the structure electrodeposited on the surfaces is also relatively uniform, which are similar to the A-20 sample. In addition, the enlarged image on the right shows that the superhydrophobic structure is also composed of a large number of interlaced nanorods with a breadth around 50 nm.

1.3 Evaluation of static icing behavior

1.3.1 Nucleation Process of Ice

During the heterogeneous nucleation process of ice under the same temperature condition, the energy compensation provided by the substrate for nucleation can be expressed as:

$$W_{hetro}^{*}=\frac{16\pi\sigma_{IS}^{3}v_{I}^{2}}{3\Delta\mu_{IS}^{2}}\varphi(\theta) \quad (1)$$

Where

$$\varphi(\theta)=\frac{(1-\cos\theta)^{2}}{4}(1+\cos\theta) \quad (2)$$

Here W_{hetro}^{*} indicates the energy compensation for the formation of a nucleus of ice in substrate in case of heterogeneous nucleation, σ_{IS} is the surafce energy of the substrate interface, v_I is the molar volume of ice, $\Delta\mu_{IS}=v_I(p_S-p_I)$ is the difference between the chemical potentials of molecules in water molecule and substrate if the thermodynamic state of substrate remains unchanged during nucleation. The (p_S-p_I) is the pressure

difference between the two phases, and the coefficient $\varphi(\theta)$ is determined by water contact angle on substrate. It can be considered that the decrease of surface energy can reduce the energy compensation obtained from the substrate, and then increases the demand for nucleation energy provided by the external system, resulting in the delay of the icing process.

Generally, the influence of surface energy on anti-icing performance is limited. We achieved superhydrophobic surfaces with similar micro-nanostructures but different surface energies on the same aluminum plate by controlling the carbon chain length of organic acids ($[CH_3(CH_2)_{12}COO]_3Ce$ and $[CH_3(CH_2)_{14}COO]_3Ce$, where the longer the carbon chain, the lower the surface energy). The corresponding surface topography is shown in Supplementary Fig. 9.

The anti-icing experiment shows that there is no obvious discrepancy in the icing delay time of superhydrophobic surfaces with different surface energies, as shown in Supplementary Fig. 10. However, for the superhydrophobic surfaces with different microstructure in this work, even if the surface energy is consistent, the icing delay time of sample A-30 is 800 s-900 s higher than that of other samples. This indicates that the surface structures modification is more important for anti-icing process.

In this work, considering the geometric characteristics of the arrayed microstructure on the superhydrophobic surface, the larger the structural angle, the smaller the space occupied by droplets within the microstructure, as shown

in Supplementary Fig. 11. The microstructure with a larger angle can retain more micro-air-pockets to hinder temperature transfer, leading to an improvement of anti-icing performance. This is why the anti-icing performance of A-20 sample is lower than that of A-30 sample.

However, for droplets with fixed size, the larger the structural angle, the more solid-liquid contact interfaces within the same contact radius. Excessive structural angle tends to easily cause a wide range of direct temperature transfer from the low-temperature substrate, promoting the nucleation of droplets. Therefore, there was an abnormal decrease in the icing delay time on A-40 sample.

1.3.2 Wettability and Mobility of Superhydrophobic Surface Under Different Temperature

The wettability and mobility of the various superhydrophobic surface with nanostructure have been examined and compared under different temperature conditions. The water contact angle (WCA), sliding angle (SA) and droplet morphology images are presented in Supplementary Fig. 12. It can be observed that the flat surface with nanostructures exhibits super-hydrophobicity at room temperature (a WCA of 160.62° and SA of 2.8°). Meanwhile, all three hierarchical structure surfaces demonstrate excellent super-hydrophobicity after combining this nanostructure with the array microstructure (the WAC/SA of A-20, A-30 and A-40 samples are 169.74°/1.9°, 170.82°/1.7° and 167.31°/2.1°, respectively). Subsequently, the CAs of all these

superhydrophobic surfaces are decreased to about 145° when the temperature is decreased to 273.15 K, while the SAs of these surfaces are unable to obtain due to the wetting between the droplets and the substrate with condensate water. Additionally, the CAs of superhydrophobic surfaces further reduce to around 130° when the ambient temperature drops to 263.15 K. Notably, the superhydrophobic flat surface shows a lower CA of 124.45° at 263.15 K. However, the variation of CA on hierarchical structure surfaces is not obvious as the temperature further decreases to 253.15 K, nevertheless, the CA of the flat surface is reduced by 6°. This indicates that simple nanostructures are difficult to retain the air-pockets captured on the surface in low-temperature environments. Moreover, except for the A-30 sample, the droplets freeze on the surfaces within a few seconds when the temperature drops to 243.15 K. Furthermore, the droplets freeze immediately upon contact with all the superhydrophobic surface when the temperature decreases to 233.15 K. Generally, the existence of arrayed microstructures can effectively enhance the water repellency of superhydrophobic surfaces in low-temperature environments.

Moreover, water molecules at the solid-liquid interface inevitably freeze and nucleate with the decrease of temperature. This leads to the transition of the contact interface from water-solid to water-ice. Meanwhile, the pressure of the air-pocket inside the micro-nanostructure of the superhydrophobic surface can also be reduced as the temperature drops, weakening the support effect of

the superhydrophobic surface, and causing an enlargement of solid-liquid interface. Therefore, under the coordination of the above two mechanisms, the transition from Cassie to Cassie-Wenzel also happens on all the samples.

1.3.3 Icing Delay Performance

It is worth noting that the icing delay ability of the three samples decreases sharply when the surface temperature is reduced to 243.15 K, as shown in Supplementary Fig. 13. Even for the A-30 sample with a superior icing delay performance, its icing delay time is shortened to 33 s, which is 29 s longer than that of the superhydrophobic plate. Meanwhile, the icing delay times of A-20 and A-40 samples are even reduced to 2-3 s, which is only 6%~9% of that of A-30 sample, indicating a loss of icing delay ability. With the temperature is further decreased to 233.15 K, although the A-30 sample still exhibits a longer icing delay time, the droplets on all three surfaces start to freeze within 5 s. This demonstrates that icing behavior is hard to postpone effectively by these three samples at 233.15 K. Astoundingly, the time required for the droplet to freeze completely on the A-20 sample is always longer while the time required for A-40 sample is shorter under all experiment temperature, when the contact region between the droplet and the sample has already been frozen. This discrepancy may arise from variations in the solid-ice interfacial area among the different surfaces. The ice is inevitably intruded into the microstructure during freezing process. The larger the angle of arrayed microstructure, the more the number of microstructures. In regions with the same coverage, A-40 sample with more

microstructure acquires larger contact interface between the substrate and the ice, thereby facilitating the complete icing process.

The superhydrophobic A-30 sample can still maintain lower ice accumulation on the surface at 243.15 K and 233.15 K, respectively, as shown in Supplementary Figs. 14 and 15. It is despondent that A-40 sample does not exhibits effective anti-icing ability under any temperature conditions after superhydrophobic treatment.

1.4 Analysis of static icing process of microdroplet

The free energy of ice is always lower than that of water under supercooled conditions, hence, there has been a sudden drop in system energy when water molecules start to nucleate. The total potential energy data show that there are four stages in the freezing process[2]: (1) a long quiescent period with relatively constant potential energy; (2) a short period during which the potential energy slowly decreases; (3) a short period during which the potential energy decreases rapidly; and (4) a final period with reduced but relatively constant potential energy and during which the ice structure fully forms. Water in the quiescent period is in a supercooled liquid state, exhibiting intermittent collective motions and energy fluctuations associated with hydrogen bond rearrangements. The freezing process starts in stage (2). The fact that the system explores the overall relatively flat potential energy landscape for a considerable time (that is, the quiescent period) before entering the fast-growing period agrees with the predictions of basic nucleation theory[3,4,5].

However, the MD simulation also provides a molecular-level illustration of the water freezing process not obtainable from conventional nucleation theory[5]. The corresponding nucleation temperature and time can be achieved from Supplementary Fig. 16.

Additionally, ice tends to nucleate and grow rapidly along easy growth direction, and the change of growth orientation is to adapt to the limitation of nucleation space. This change will disrupt the original growth inertia of ice, hindering the growth of the ice layer along the easy growth direction, resulting in a delay in nucleation and growth.

Notably, almost no regular ice crystal can be observed on A-30 sample, and the cubic ice and hexagonal ice is not arranged in a layered form, demonstrating a chaotic distribution of ice crystal (this confusion is more chaotic than the cubic/hexagonal hybrid structure). This may be due to the spatial limitations of the icing environment that cause the ice crystals to constantly adjust their orientation during nucleation. This adjustment requires longer time for the nucleation and growth of ice crystals, exhibiting an icing behavior with multi-orientation, and delaying the ice process.

Moreover, the limited growth space is mismatched with the ice type structure due to the unique spatial structure and growth orientation of cubic ice and hexagonal ice, as illustrated in Supplementary Fig. 17. Ice formation is the process in which water molecules fill the space without creating vacancies as much as possible. Due to the mismatch between ice and substrate, cubic ice

and hexagonal ice are always forced to mix and adjust their orientation to achieve space filling. It is obvious that hexagonal ice tends to grow laterally rather than at the top due to its anisotropy. This growth orientation often promotes the formation of hexagonal ice with chain structures, making it difficult to form tight structures in confined environments at atomic level[6].

The detailed nucleation process of A-20 and plate models under the surface interaction energy of 1.18 kcal mol^{-1} is shown in Supplementary Fig. 18. It is clear that the regular structure with the same orientation gradually extends to the whole ice layer on both A-20 sample and the plate sample with the further extension of icing time. Moreover, almost no regular arrays of water molecules with the same orientation have been observed in any direction other than the front view.

1.5 Prediction of dynamic motion behavior for microdroplet

The movement of microdroplets on A-20 sample is also calculated using Fluent Icing method, as shown in Supplementary Fig. 19. It is clear that the heat exchange at the terminus is gradual less than that at the forepart, as shown in Supplementary Fig. 19a. A lot of areas inside the microstructure (as marked by red) have an inverted heat transfer identically. Moreover, liquid water content (LWC) analysis shows that the range of low LWC near the substrate gradually rises along the flow direction, and the LWC within the subsequent microstructures are maintained at an extremely low level, as shown in Supplementary Fig. 19b. The LWC data extracted from the test area

demonstrates that the LWC above the microstructure is sharply decreased to a lower value with the increase of microstructure quantity, as shown in Supplementary Fig. 19c. Subsequently, droplet collection coefficient image reveals that microdroplets are always attracted to the front of the microstructure sample, as shown in Supplementary Fig. 19d. Similarity, the microdroplets are basically gathered at the top of the subsequent microstructure with a decrease tendency when the microstructures gradually increase in the flow direction, as shown in Supplementary Fig. 19e. The ice distribution results in Fig. 19f confirm that the ice accumulation on the microstructure surface is mainly distributed at the front of the sample, and even several microstructures that are almost not covered by ice appear at the terminal of the sample.

The icing mass curves of plate and A-30 sample are illustrated in Supplementary Fig. 20. It can be seen from the figure that the variation of icing mass is highly consistent with the distribution of droplet collection coefficient. In addition, higher droplet collection coefficient values on sample A-30 also lead to greater icing mass. Although a large number of non-icing areas appear on the subsequent microstructure, it is still difficult to make up for the accumulation of excessive microdroplets on the front structure of A-30 sample, resulting in a higher overall icing quality.

1.6 Icing behavior of microdroplet in icing wind tunnel

1.6.1 Ice Accumulation and Microdroplet Movement on A-20 Sample

The icing wind tunnel test also carried on the A-20 sample with the flow

velocity of 69.4 m s^{-1}, as shown in Supplementary Fig. 21. Analogously, the ice on the A-20 surface shows a decrease tendency along the flow direction. After superhydrophobic treatment, the ice accumulation on the A-20 surface is distributed evenly and sparsely. Compared to the conventional superhydrophobic plate, the A-20 surface with superhydrophobic treatment also achieves a lower ice accumulation, which is 33.7%~39.2% lower than that accumulated on the superhydrophobic plate. This also verifies that the arrayed aerodynamic microstructures can effectively improve the anti-icing properties of superhydrophobic materials under high speed condition.

The microdroplet movement on the A-20 surface is also measured by high-speed camera, as shown in Supplementary Fig. 22. The shape of the microstructure in these figures has changed due to the difference in observation angles, as indicated by the red dotted line in Supplementary Fig. 22a. The results show that the microdroplets could bounce off the surface easily at room temperature and 263.15 K, as displayer in Supplementary Figs. 22b-c. Moreover, it is also observed that some microdroplets can successfully bounce off the surface when the temperature is reduced to 253.15 K, as illustrated in Supplementary Fig. 22d. However, the phenomenon of microdroplets rolling on the top of the microstructures is observed for the first time under the same condition, as demonstrated in Supplementary Fig. 22e. This is due to the fact that the micro-vortexes inside the structure occupy a small area, which is difficult to provide the driving force for the droplet to completely leave the

surface. The microdroplets tend to sink due to the lack of airflow support when they roll over the micro-vortex. Subsequently, these microdroplets are then lifted up again by touching the next micro-vortex, causing the droplets to roll on top of the microstructure. This also verifies that the micro-vortex inside the microstructure can reduce the contact between the microdroplet and the surface.

1.6.2 The Influence of Incident Angle on Anti-Icing Performance

Considering the directionality of the drag-reduction microstructure, A-20 and A-30 samples are selected for anti-icing experiment in the icing wind tunnel with the incident angles of 0°, 10°, 20°, 30°, 40°, 50°, 60°, 70°, and 80° respectively, in order to investigate the effect of incident angle on the anti-icing performance of the arrayed hierarchical structure, as illustrated in Supplementary Fig. 23a. The flow velocity is set as 69.4 m s^{-1} and the temperature is defined as 253.15 K, which is consistent with the previous anti-icing test.

The results indicate that the ice accumulation on all samples increases with the decrease of incident angle, as shown in Supplementary Fig. 23b-j. Similar to the previous results, the ice accumulation on the end of the superhydrophobic plate is still higher than that on the front, which is determined by the motion behavior of microdroplets driven by interfacial air flow. Meanwhile, a certain amount of ice accumulation also exhibits on the end of the arrayed hierarchical structure due to the appearance of incident angle. Notably, the ice

accumulation on the two arrayed hierarchical structures is lower than that on conventional superhydrophobic surfaces (the ice accumulation reduction is about 30%) at a higher incident angle (>40°), demonstrating effective anti-icing applicability, as shown in Supplementary Fig. 24. With the incident angle further reduces to 40°, the ice accumulation reduction of A-20 sample significant decreases to -29.6%. Subsequent results indicate that although the A-20 sample completely loses its superiority in anti-icing when the incidence angle is below 20°, nevertheless, its anti-icing property is again reflected with the further descension of incident angle, indicating the widespread applicability of the array hierarchical structure in low-temperature and high-velocity inflow environments.

2. Supplementary Method

2.1 Design, fabrication and verification of microstructure

The microstructural unit of the barchan dune was inspired by the regularly arranged natural barchan dune, which was consist of unique windward and slip surfaces, indicating a strong ability to maintain shapes[7,8,9,10,10,12]. In this work, a symmetrical geometric shape was extracted from the cross section of barchan dune to con-struct the microstructure unit, as proposed in our previous work[13]. The computational domain used in this work is the same as that used in the previous work, as shown in Supplementary Fig. 25[13].

The numerical simulation was implemented by Ansys Fluent 2022 in 2D model. For in-compressible fluid with velocity below 0.3 Ma, a pressure-based solver was used in this work. Both the TOP and BOTTOM regions were set as symmetry boundary in order to prevent the interference from side walls, as depicted in Supplementary Figure 25. In addition, the PLATE and STRUCTURE regions were defined as stationary walls with no slip. The INLET and OUTLET regions were set as velocity inlet boundary and pressure outlet boundary respectively under incompressible flow condition.

Considering the aeronautical applications, the flow velocity (U_∞) was defined as 69.4 m s^{-1}. The Reynolds number of a plate was defined as following:

$$\mathrm{Re}_L = \frac{U_\infty L}{\nu} \tag{3}$$

where L is the distance from the calculation position to the inlet of flow field, U_∞ is the free flow velocity, and ν is the kinematic viscosity coefficient.

The kinematic viscosity coefficient of air is 1.4607× 10^{-5} m^2 s^{-1}. The corresponding Reynolds number (Re_L) is 9.50× 10^5, which means a turbulent state in flow field.

Reducing the burst in the turbulent boundary layer is an effective way to reduce drag. Therefore, the height of the microstructure should be smaller than the boundary layer thickness (δ_L) in order to regulate the drag by disturbing the boundary layer. Meanwhile, the depth of the microstructure unit also should be greater than allowable roughness values (k_{adm}) to ensure that the surface is beyond the scope of dynamic smoothness in hydromechanics. To determine whether the microstructure can affect the turbulent boundary layer, the δ_L and the k_{adm} can be determined by following formulas[1]:

$$\delta_L = 0.37L \times \mathrm{Re}_L^{-0.2} \tag{4}$$

$$\mathrm{k}_{adm} \le \frac{100L}{\mathrm{Re}_L} \tag{5}$$

According to the flow velocity applied in this work, the allowable roughness value was 21.65 μm. Thus, the height of microstructure could be set as 40 μm, 50 μm, 60 μm, 80 μm, 100 μm, 120 μm and 140 μm, respectively. Mean-while, on account of the angle (α) processed a typical value of around 30° in nature, the α was defined as 20°, 25°, 30°, 35° and 40°, respectively.

Previous research indicated that the realizable k-ε model was suitable to describe the boundary layer thickness with high precision[14,15]. Additionally, this model could minimize the error between simulation and experiment[16]. The comparison between the theoretical values and the calculated values under

different turbulence models also verified the suitability of the realizable k-ε model, as shown in Supplementary Fig. 26. Therefore, the underlying physical mechanism of drag reduction induced by micro-structure was studied by the realizable k-ε model in this work.

The governing equation could be referred to previous literatures as follows[17,18]:

The realizable k-ε model:

$$\frac{\partial}{\partial t}(\rho k) + \frac{\partial}{\partial x_i}(\rho k u_j) = \frac{\partial}{\partial x_i}\left[\left(\mu + \frac{\mu_t}{\sigma_k}\right)\frac{\partial k}{\partial x_j}\right] + G_k + G_b - \rho\varepsilon - Y_M + S_k \tag{6}$$

$$\frac{\partial}{\partial t}(\rho\varepsilon) + \frac{\partial}{\partial x_j}(\rho\varepsilon u_j) = \frac{\partial}{\partial x_j}\left[(\mu + \frac{\mu_t}{\sigma_\varepsilon})\frac{\partial \varepsilon}{\partial x_j}\right] + \rho C_1 S\varepsilon - \rho c_2\frac{\varepsilon^2}{k+\sqrt{\nu\varepsilon}} + C_{1\varepsilon}\frac{\varepsilon}{k}C_{3\varepsilon}G_b + S\varepsilon \tag{7}$$

Where

$$C_1 = max\left[0.43, \frac{\eta}{\eta+5}\right] \tag{8}$$

$$\eta = S\frac{k}{\varepsilon} \tag{9}$$

$$G_k = \mu_t S^2 \tag{10}$$

$$S = \sqrt{2S_{ij}S_{ij}} \tag{11}$$

$$S_{ij} = \frac{1}{2}(\frac{\partial u_j}{\partial x_i} + \frac{\partial u_i}{\partial x_j}) \tag{12}$$

$$Gb = -g_i\frac{\mu_t}{\rho Pr_t}\frac{\partial \rho}{\partial x_i} \tag{13}$$

For ideal gas, the turbulent viscosity coefficient is expressed as following

$$\mu t = \rho C\mu\frac{k^2}{\varepsilon} \tag{14}$$

The enhanced wall treatment, which was suitable for complex flow in a high-Reynolds-number turbulence model, was used for the near-wall treatment. More specific parameters of above equations can be referred to previous literatures[17,18].

The previous solver parameters and grid construction methods were followed in this work[13]. Moreover, the independence of the computational grid was also verified to ensure the accuracy of the calculation, as shown in Supplementary Fig. 27.

Several velocities were chosen to verify the dependability of the calculation model, and the theoretical skin friction coefficient was compared with the simulation result. The theoretical value of skin friction coefficient (C_f) was expressed as $C_\mathrm{f} = 0.074 \times \mathrm{Re}_L^{-0.2}$. As listed in Supplementary Fig. 28, the theoretical skin friction coefficient value is decreased from 4.713 × 10^{-3} to 4.106 × 10^{-3} with the increase of flow velocity under above boundary conditions. Otherwise, simulation results demonstrate the same tendency with theoretical values. The maximum relative error between the simulation and theoretical results is only 6.99%, indicating the reliability of calculation model used in high speed flow condition. Moreover, additional simulations with a top boundary of wall with a velocity gradient of 0 and a bottom boundary of no slip were implemented to further check the numerical accuracy of the results. The difference between the theoretical value and the simulation value can also be controlled within 7%, which is similar with the data obtained under symmetry boundary conditions. It can be verified that the above model still has decent reliability even under the wall boundary condition, revealing a better applicability in simulation work. Moreover, drag reduction ratio (R_D) is usually used to evaluate drag reduction performance of non-smooth surfaces. The drag reduction rate is calculated using the following equation:

$$R_D = \frac{F_{smooth} - F_{non-smooth}}{F_{smooth}} \tag{15}$$

where $F_{\text{non-smooth}}$ and F_{smooth} are the total drag (consist of skin-friction drag and pressure drag) of a model with and without microstructures region in the middle of the calculation domain.

The micro-milling method is used to prepare the designed microstructure on the aluminum alloy surface. A diamond cutter with a tip diameter of 1 μm is used for top-down milling.

A vertical circulating wind tunnel with a resistance sensor is used to simulate the change of resistance on the sample during real flight, as shown in Supplementary Fig. 29. The flow velocity is set at 69.4 m s^{-1}. The observation section of the wind tunnel is transparent to facilitate subsequent observation of the sample under high-velocity fluid conditions, and the sample is clamped horizontally in the middle of the observation section, as shown in the enlarged area.

2.2 Preparation and characterization of superhydrophobic surface

Before the electrodeposition process, the specimens were polished with a series of emery papers (from 240# to 2000#), then mechanically polished to smooth, therewith, cleaned ultrasonically in acetone (acquired from Sinopharm Chemical Reagent Co., Ltd., China. the purity is about 99.5%), ethanol (acquired from Sinopharm Chemical Reagent Co., Ltd., China. the purity is about 99.5%) and deionized water (generated in our laboratory) for 30 min respectively, as shown in Supplementary Fig. 30. On account of different

electrolytic solutions, the electrodeposition processes were performed on a direct current power with a series of constant voltage and time at different temperature, where the specimen was used as the cathode and the platinum plate as the anode. The distance of the two electrodes was defined as 2 cm. Cerium nitrate hexahydrate ($Ce(NO_3)_3{\cdot}6H_2O$, 0.0002 mol L^{-1}, obtained from Shanghai Aladdin Biochemical Technology Co., Ltd. the purity is about 99.95%) and the Stearic acid (0.0008 mol L^{-1}, obtained from Shanghai Aladdin Biochemical Technology Co., Ltd. the purity is about 99.5%) immersed in ethanol were used as the electrolyte solution. The electrodeposition temperature of the process was 313.15 K. In addition, the volume of the electrolyte solution was set as 100 mL. After deposition, the sample was carefully removed from the electrolyte and rinsed thoroughly with ethanol, thereafter dried at 353.15 K on the hotplate for 24 h.

The morphological and elementary composition of the electrodeposited surfaces were performed using a field emission scanning electron microscope (FE-SEM, Hitachi S4800, Japan) equipped with energy dispersive X-ray spectroscopy (EDS). The chemical composition was analyzed by a combination of X-ray photoelectron spectroscopy (XPS, Thermo Scientific K-Alpha) using a corrected spectral line of C1s at 284.6 eV and Fourier transformed infrared spectroscopy (FT-IR, Nicolet IN10, ThermoFisher) with a resolution of 4 cm^{-1}. The phase analyses on the thinner electrodeposited surfaces were conducted by a Grazing Incidence X-ray diffractometry (GIXRD, D8 ADVANCE Cu Kα

radiation, Bruker) with a fixed incident angle of 0.1°.

The static non-wettability of the electrodeposited surfaces was reflected by CA and CSA, which was measured by a contact angle analyzer (Kruss DSA100, Germany). For all surfaces, at least five repetitions of measurement were performed at independent locations with a water droplet around 4 μL, and the values were statistically averaged to ensure the accuracy of the experimental data.

2.3 Evaluation of icing delay behavior

Numerous microdroplet freezing tests were conducted in a self-made icing environment system including a microdroplet generator and a constant temperature and humidity chamber, as observed in Supplementary Fig. 31. In order to prevent the measurement error caused by residual water on the surface, the microdroplet generator was placed far below the cold table to slowly release the microdroplet and maintain the ambient humidity of 100%. Meanwhile, the samples and microdroplets was synchronously cooled by the ambient temperature controlled by the temperature and humidity chamber. Moreover, in order to avoid the interference of icing on other surfaces to the statistics, the surface other than the test surface (top) was covered with a mask. When the mask was removed, the mass difference of the sample before and after the microdroplets dispersed to the surface and freeze was defined as the icing mass.

2.4 Molecular dynamics analysis of static icing process

In this work, we aim to survey the icing behavior of microdroplets under high velocity inflow conditions. A large amount of microdroplets that impact the superhydrophobic surface can easily break away from the surface before ice nucleation with the assistance of the wind field. However, due to the disordered distribution and scale difference of incoming microdroplets, partial microdroplets can always enter the microstructures and freeze at the bottom, which is similar to a static icing process. Notably, the microdroplet is possible to detach from the superhydrophobic surface again under airflow disturbance when the microdroplet fails to freeze immediately after entering the structure. Therefore, it is necessary to clarify the influence of microstructure on the static icing behavior of droplets in order to accurately analyze the effect of structural angle on the final anti-icing property.

Previous literature has shown that the angle of microstructure has a significant influence on the static freezing behavior[19]. Although the settings of molecular dynamics simulation and experiment are not strictly the same, MD simulation is not used to verify the icing experiment in this work, but the underlying mechanism of angle on static icing behavior is revealed by both the experiment and MD simulation methods. Moreover, the icing process with gradual decreased temperature is adopted instead of constant cooling temperature selected in the experiment in order to save computing resources on the premise of revealing the static icing mechanism, since the icing process with constant cooling temperature requires an extremely long calculation time.

Furthermore, a new anti-icing mechanism of superhydrophobic surface under high-velocity flow condition with low temperature is proposed by considering the influence of microstructure on static icing and dynamic icing synchronously.

For the hierarchical structure with both drag reduction and superhydrophobic properties in this work, the substrate is aluminum alloy and the surface are covered with nanoscale cerium stearate. The water repellency of superhydrophobic surface is mainly caused by the low surface energy of cerium stearate molecules and the micro-nanostructure formed by the agglomeration of cerium stearate molecules. Although the low surface energy of cerium stearate molecules can be directly reflected in MD simulation by constructing cerium stearate molecular chains, the micro-nanostructure with randomness on the surface is difficult to realize by MD simulation. Therefore, the superhydrophobic properties of the actual superhydrophobic samples are approximated by assigning surface interaction parameters.

The molecular dynamics simulation software of LAMMPS was used to calculate the nucleation process of water molecules on the typical microstructure surfaces. The microstructure is reduced proportionally with a height of 1.5 nm in order to ensure the reliability of modeling and save computing resources. Meanwhile, the thickness of water molecular layer was set as 4 nm to exclude the influence of thickness on nucleation icing. The corresponding number of water molecules were set as 9900, 17600 and 9870 on the plate, microstructure surface with angle of 20° and microstructure

surface with angle of 30°, respectively. After the atomic coordinates of the substrate are fixed and the system is minimized by energy, kinetic relaxation of 40 ps is performed at the temperature of 298 K to allow liquid water to enter the structure and complete the preparation for modeling.

The models are located at the bottom of the simulation box, and a vacuum layer with a height of 400 nm is set above the models, which can completely eliminate the boundary effect of the periodic boundary[20], as shown in Supplementary Fig. 32.

Considering the influence of gravity in the actual freezing environment, the gravity of 6.24×10^{-4} kcal mol^{-1} $Angstrom^{-1}$ was applied in the simulation process. Since the position of the substrate atoms is fixed, there is no need to set up interactions between the substrate atoms. The interaction between water molecules adopts mW coarse-grained potential. The mW is a common using monatomic water model, and the mW model of water does not have hydrogen atoms or electrostatics. Water molecules are represented as a single particle and able to form tetrahedral "hydrogen-bonded" structures through three-body nonbonded interactions. The interactions between mW water molecules consist of the sum of pairwise and three-body contributions described by the functional form of the Stillinger−Weber (SW) potential:

$$E=\sum_{i}\sum_{j>i}\phi_2(r_{ij})+\sum_{i}\sum_{i\neq j}\sum_{k>j}\phi_3(r_{ij},r_{ik},\theta_{ijk}) \tag{16}$$

$$\phi_2(r_{ij})=A_{ij}\varepsilon_{ij}\left[B_{ij}\left(\frac{\sigma_{ij}}{r_{ij}}\right)^{p_{ij}}-\left(\frac{\sigma_{ij}}{r_{ij}}\right)^{q_{ij}}\right]\exp\left(\frac{\sigma_{ij}}{r_{ij}-a_{ij}\sigma_{ij}}\right) \tag{17}$$

$$\phi_3\left(r_{ij}, r_{ik}, r_{ijk}\right) = \lambda_{ijk}\varepsilon_{ijk}\left[\cos\theta_{ijk} - \cos\theta_{0ijk}\right]^2 \exp\left(\frac{\gamma_{ij}\sigma_{ij}}{r_{ij} - a_{ij}\sigma_{ij}}\right)\exp\left(\frac{\gamma_{ik}\sigma_{ik}}{r_{ik} - a_{ik}\sigma_{ik}}\right) \quad (18)$$

where φ_2 is a two-body term and φ_3 is a three-body term. The summations in the equation are over all neighbor atoms of a certain atom within a cutoff distance. In our simulation, it just needs to set up the ε = 6.189 kcal mol^{-1}, and σ = 2.3925Å to get mW water model.

Additionally, LJ potential is used to define the force between water molecules and substrates atoms[21]. LJ potential is described by following equation:

$$\mathrm{E} = 4\varepsilon\left[\left(\frac{\sigma}{r_{ij}}\right)^{12} - \left(\frac{\sigma}{r_{ij}}\right)^{6}\right] \quad (19)$$

Afterwards, to survey the influence of surface energy (ε_{ws}) variation on icing behavior, different interaction energies are inflicted on the surface. Therein, the contact angles of water droplets are verified under different surface interaction energy conditions, as shown in Supplementary Fig. 33. In this work, the interaction energy of flat surface is set as 0.12 kcal mol^{-1} so that its contact angle is about 160°, which is similar to the actual contact angle of the flat plate after superhydrophobic treatment for 10 min. Hence, the ε of 0.12 kcal mol^{-1} used to describe the icing behavior on superhydrophobic surface is considered credible in this work. Meanwhile, the surface energy of pure aluminum is 1.18 kcal mol^{-1}. As a comparison, the surface energy of graphene of 0.21 kcal mol^{-1} is also adopted, and the detailed interaction parameters between water and substrate are set in the Supplementary Table 1.

Moreover, three-dimensional periodic boundary conditions were selected for the simulation system. the relevant literature shows that the ramps are performed with cooling rates of 5 K ns^{-1}, 2 K ns^{-1}, and 1 K ns^{-1}, and only the latter resulted in crystallization of ice[22]. Hence, the cooling rate of 1 K ns^{-1} is adopted in this work. Additionally, the above literature also reveals that the equations of motion of water are integrated with the velocity Verlet algorithm with a time step 5 fs in the case of the systems with an open water/vacuum interface and 10 fs for the bulk systems[22]. Meanwhile, the calculation time step can be extended appropriately due to the employment of coarse granulation potential. On this basis, we believe that a time step of 5 fs is acceptable for this calculation. Hence, the nucleation and growth process were investigated during cooling simulation which temperature changed from 290 K to 180 K with cooling rate of 1 K ns^{-1}. The Nose method was used to control temperature which is a suitable cooling rate for observing the nucleation process[22]. Simulations were performed in the NVT ensemble and the equations of water motion were integrated with the velocity Verlet algorithm with a time step of 5 fs.

Furthermore, the ice nucleation is the ordering process of disordered water molecules. The icing process can be captured by monitoring the structure of water molecules[23]. Water molecules in ice are usually arranged in a cubic or hexagonal structure, and the ice structure can be identified using a molecular visualization software OVITO. The recognition path of this software is as follows[24]: First, the nearest neighbors of an atom are identified. Then, for each

of these four neighbors, their respective nearest neighbors are identified. This yields the list of second nearest neighbors of the central atom. Finally, the CNA fingerprint is computed for these 12 second nearest neighbors and the central atom. If they are arranged on an FCC lattice. then the central atom is classified as cubic diamond. If they form an HCP structure. then the central atom is marked as a hexagonal diamond atom.

2.5 Impact and icing behavior of microdroplet under low temperature

In the process of solving microdroplets movement through CFD, the air and micro-droplets could be solved simultaneously (as a two-phase flow). However, since the micro-droplets volume fraction is very small, the two-phase flow is considered a dilute gas-particle flow and thus the governing equations of air and micro-droplets are solved in a segregated manner. The airflow is solved first, followed by the micro-droplet equations. In this manner, the effect of the air on the micro-droplets is considered.

In this work, SST-K-ω model is adopted to describe the flow field. For icing simulation, the non-dimensional k and ω on a wall can be expressed as[25]:

$$k_{\omega}^{+} = \max\left\{0, \frac{1}{\sqrt{\beta^{*}}}\tanh\left[\left(\frac{\ln\frac{h_s^{+}}{30}}{\ln 10} + 1 - \tanh\frac{h_s^{+}}{125}\right)\tanh\frac{h_s^{+}}{125}\right]\right\} \tag{20}$$

$$\omega_{w}^{+} = \frac{300}{h_s^{+2}}\left(\tanh\frac{15}{4h_s^{+}}\right)^{-1} + \frac{191}{h_s^{+}}\left[1 - e^{\left(-\frac{h_s^{+}}{250}\right)}\right] \tag{21}$$

where β^{*}=0.09, y^{+} and h_s^{+} are defined as:

$$y^{+} = \frac{\rho u_{\tau} d_{w}}{\mu} \tag{22}$$

$$h_{s}^{+} = \frac{\rho u_{\tau} h_{s}}{\mu} \tag{23}$$

Therefore, all the wall values of k and ω are known:

$$k_{w} = f_{w}\left(u_{\tau}, k_{w}^{+}\right) = k_{w}^{+} u_{\tau}^{2} \tag{24}$$

$$\omega_{w} = f_{w}\left(u_{\tau}, \omega_{w}^{+}\right) = \frac{\rho \omega_{w}^{+} u_{\tau}^{2}}{\mu} \tag{25}$$

Moreover, the flow field is modeled by partial differential equations for the conservation of mass, momentum and energy. The conservation of mass for a compressible flow, for example one where the density of the fluid is not a linear function of both pressure and velocity, can be written as:

The Energy Equation can be expressed as follow:

$$\frac{\partial \rho_{a} E_{a}}{\partial t} + \vec{\nabla} \bullet \left(\rho_{a} \vec{V}_{a} H_{a} \right) = \vec{\nabla} \bullet \left(k_{a} \left(\vec{\nabla} T_{a} \right) + v_{i} \tau^{ij} \right) + \rho_{a} \vec{g} \vec{V}_{a} \tag{26}$$

Where E and H are the total internal energy and enthalpy, ρ is the density and V is the velocity vector, the subscript a refers to the air solution. Respectively. γ is the ratio of specific heats which equals 1.4 for air (perfect gas), and k is the thermal conductivity, computed in a similar way to the laminar dynamic viscosity.

$$k = C1 \times \frac{T^{\frac{3}{2}}}{T + 133.7} \tag{27}$$

where T refers to the static air temperature in Kelvin, and where the C1 is equal to 0.00216176 W $(mK^{3/2})^{-1}$

Subsequently, the following equation is adopted to compute the dynamic

viscosity.

$$\frac{\mu_\infty}{\mu_{ref}} = \left(\frac{T_\infty}{T_{ref}}\right)^{3/2} \left(\frac{T_{ref}+110}{T_\infty+110}\right) \qquad (28)$$

where $\mu_\infty = 17.9 \times 10^{-6}$ pa·s.

The general Eulerian two-fluid model consists of the Euler or Navier-Stokes equations augmented by the microdroplets continuity and momentum equations:

$$\frac{\partial \alpha}{\partial t} + \vec{\nabla} \bullet \left(\alpha \vec{V}_d\right) = 0 \qquad (29)$$

$$\frac{\partial\left(\alpha \vec{V}_d\right)}{\partial t} + \vec{\nabla}\left[\alpha \vec{V}_d \otimes \vec{V}_d\right] = \frac{C_D \operatorname{Re}_d}{24K} \alpha \left(\vec{V}_a - \vec{V}_d\right) + \alpha \left(1 - \frac{\rho_a}{\rho_d}\right) \frac{1}{Fr^2} \qquad (30)$$

where the variables α and V_{eda} are mean field values of, respectively, the micro-droplet concentration and velocity. The first term on the right-hand-side of the momentum equation represents the drag acting on micro-droplets of mean diameter d. It is proportional to the relative micro-droplet velocity, its drag coefficient C_D and the droplets Reynolds number:

$$\operatorname{Re}_d = \frac{\rho_a d V_{a,\infty} \left\| \vec{V}_a - \vec{V}_d \right\|}{\mu_a} \qquad (31)$$

And an inertial parameter:

$$K = \frac{\rho_d d^2 V_{a,\infty}}{18 L_\infty \mu_a} \qquad (32)$$

The second term represents buoyancy and gravity forces, and is proportional to the local Froude number:

$$Fr = \frac{\left\| V_{a,\infty} \right\|}{\sqrt{L_\infty g_\infty}} \tag{33}$$

Certainly, the results of liquid water content and droplet collection coefficient have a similar presentation due to the aerodynamic characteristics of the arrayed microstructures. However, the liquid water content mainly reveals the distribution of microdroplet flow above the microstructures, while the droplet collection coefficient focuses on the probability distribution of microdroplets on the surface. Subsequently, the icing behavior of microdroplets on the structural surface is calculated based on the contact state of microdroplet.

Based on the model in Method section, icing behavior was simulated with icing module in Ansys fluent 2022 software under three-dimensional condition, as observed in Supplementary Fig. 34**.** Primarily, the flow field of these samples are need to be calculated before simulating the icing process of numerous microdroplets. Considering the accuracy and the cost of simulation, the calculation area is set to be 40 mm with 160 mm plate at both sides. When the distance between the riblet wall and the smooth plate was 20 times the microstructure height, the interactions between them could be ignored[1]. Thus, the height of this computational domain is set as 1 mm. The SST-k-w turbulence model is used to accurately capture the motion behavior of microdroplets. Both the FRONT and BACK boundaries are set as symmetry boundary in order to pre-vent the interference from side walls. In addition, the PLATE and STRUCTURE regions were defined as stationary walls with no slip. The INLET and OUTLET regions were set as velocity inlet boundary and pressure outlet

boundary respectively under incompressible flow condition. Additionally, the temperature of inlet and wall are defined as 253.15 K, while the roughness of the wall is set as 1×10^{-6} m.

Afterwards, the flow field simulation should be transmitted to Particles module. Therein, the temperature of wall is set as 265.54 K, which is 10 K higher than the Adiabatic temperature. Under Droplet conditions, set the LWC to 0.00055 kg m^{-3} and the droplet diameter to 20 μm. Under Particles distribution, keep Monodispersed since it is necessary to conduct a water catch simulation using a single droplet size. Moreover, Droplet velocity vector remains unchecked under Particles since the airflow velocity is imposed as the droplet velocity at the inlet. Subsequently, make sure that Icing model is set to Glaze under Ice accretion conditions, and leave the other settings as default.

Moreover, the independence of the computational grid is also verified to ensure the accuracy of the calculation, as shown in Supplementary Fig. 35. It can be found that the skin friction coefficient tends to be basically stable when the mesh quantity reaches 516000 for the plate model, which means that the mesh density of 250 mesh mm^{-1} is reliable.

3. Supplementary Figures

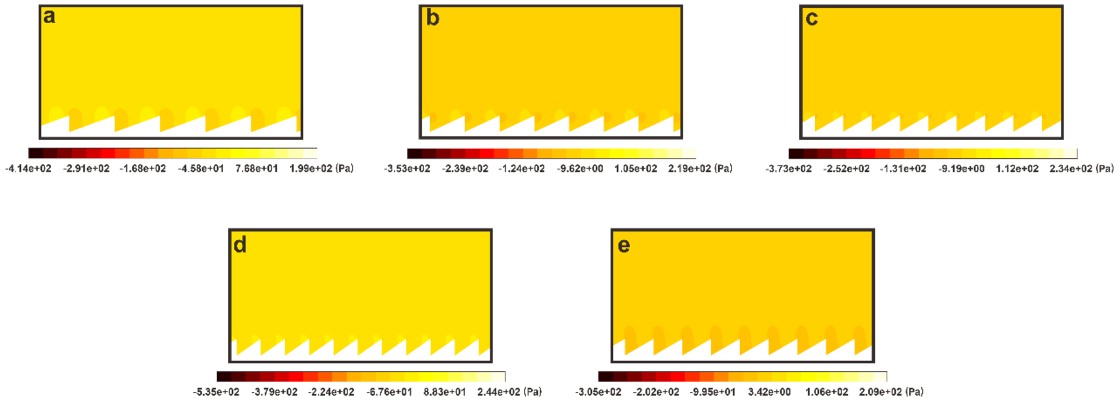

Supplementary Figure 1. **Pressure contour map of different angle of repose**.

a 20°. **b** 25°. **c** 30°. **d** 35°. **e** 40°.

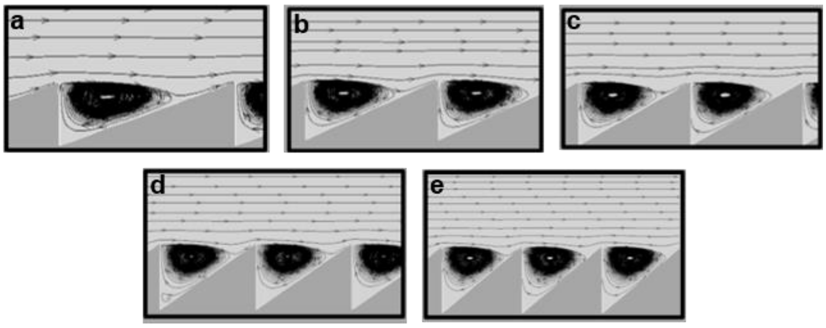

Supplementary Figure 2. **Velocity streamlines diagram of structures with different angle**. **a** 20°. **b** 25°. **c** 30°. **d** 35°. **e** 40°.

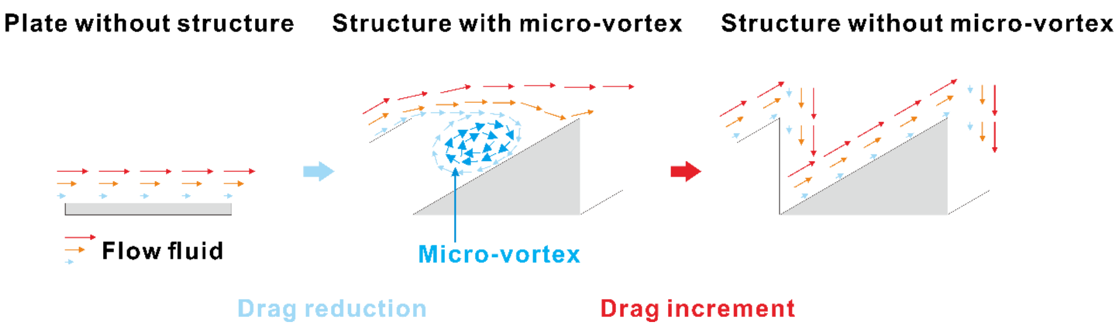

Supplementary Figure 3**. The mechanism of drag variation under high flow velocity condition.**

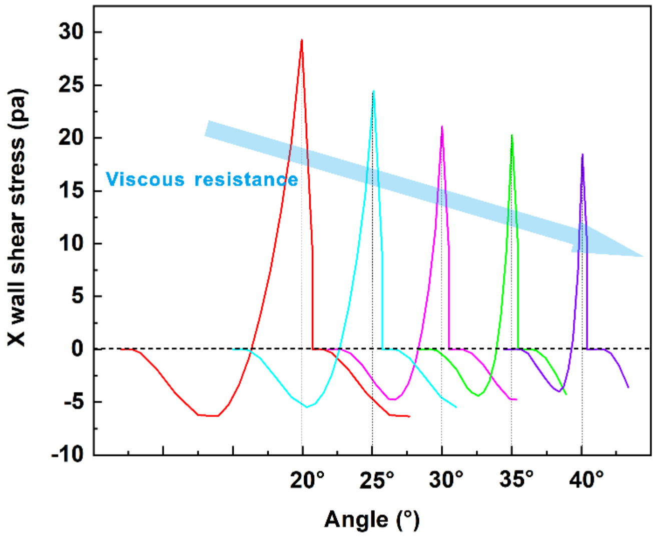

Supplementary Figure 4. **X direction wall shear stress units with different angle.**

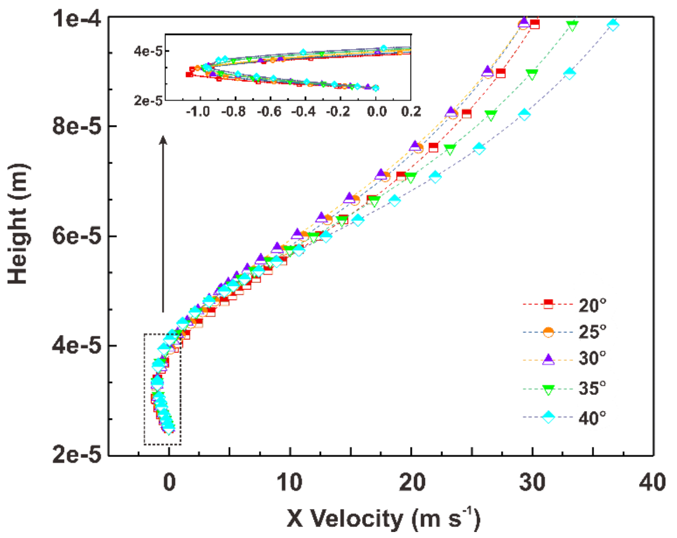

Supplementary Figure 5. **Reverse velocity gradient curves with different angle.** The reverse velocity gradient is magnified in the dashed frame.

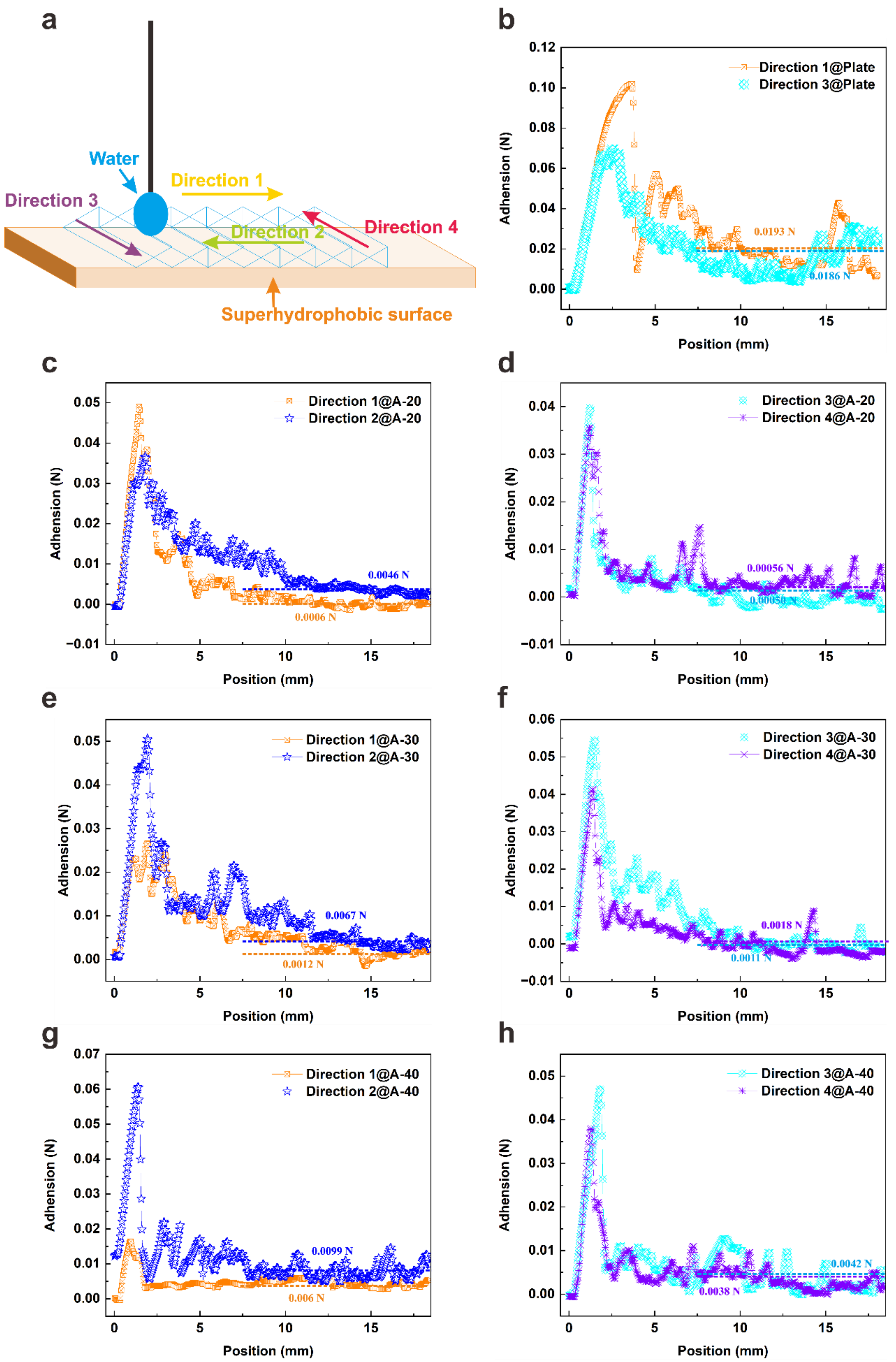

Supplementary Figure 6. **Dynamic adhesive forces of droplets on different superhydrophobic surfaces**. **a** Schematic diagram of the test process. **b**

Adhesive forces of droplets moving in directions 1 and 3 on the superhydrophobic plate. **c** adhesive forces of droplets moving in directions 1 and 2 on the superhydrophobic A-20 sample. **d** adhesive forces of droplets moving in directions 3 and 4 on the superhydrophobic A-20 sample. **e** adhesive forces of droplets moving in directions 1 and 2 on the superhydrophobic A-30 sample. **f** adhesive forces of droplets moving in directions 3 and 4 on the superhydrophobic A-30 sample. **g** adhesive forces of droplets moving in directions 1 and 2 on the superhydrophobic A-40 sample. **h** adhesive forces of droplets moving in directions 3 and 4 on the superhydrophobic A-40 sample.

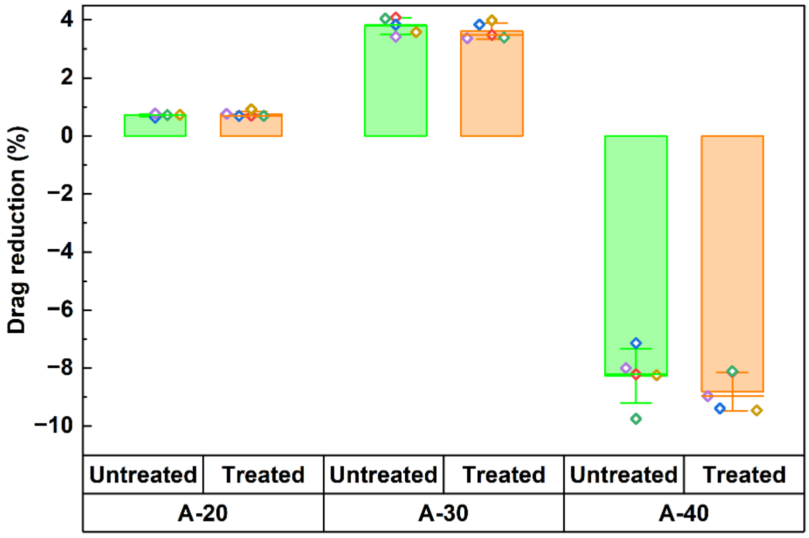

Supplementary Figure 7. **difference in drag reduction between superhydrophobic samples and untreated samples.** Error bars represent standard deviation. All the samples are tautologically measured for 5 times and averaged to mitigate potential error. The small circles in the graphs correspond to raw data.

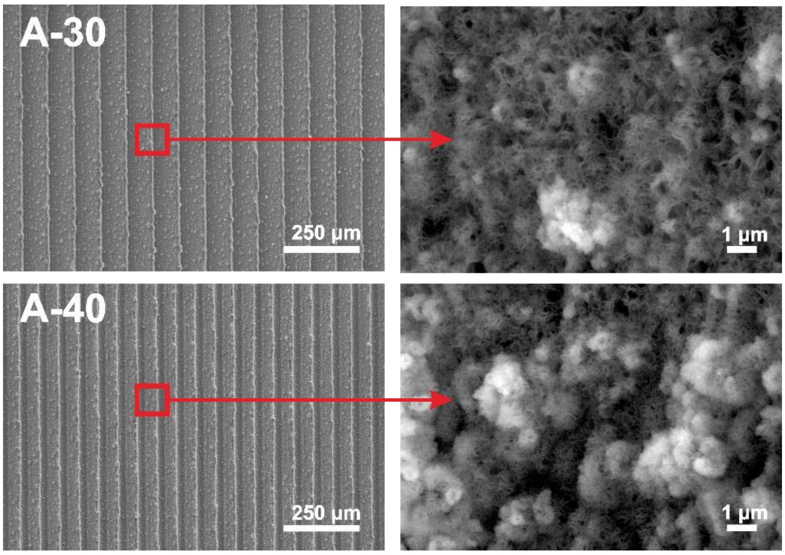

Supplementary Figure 8**. SEM images of electrodeposited surface on A-30 and A-40 samples.** The typical microstructures marked by red boxes are enlarged and displayed on the right.

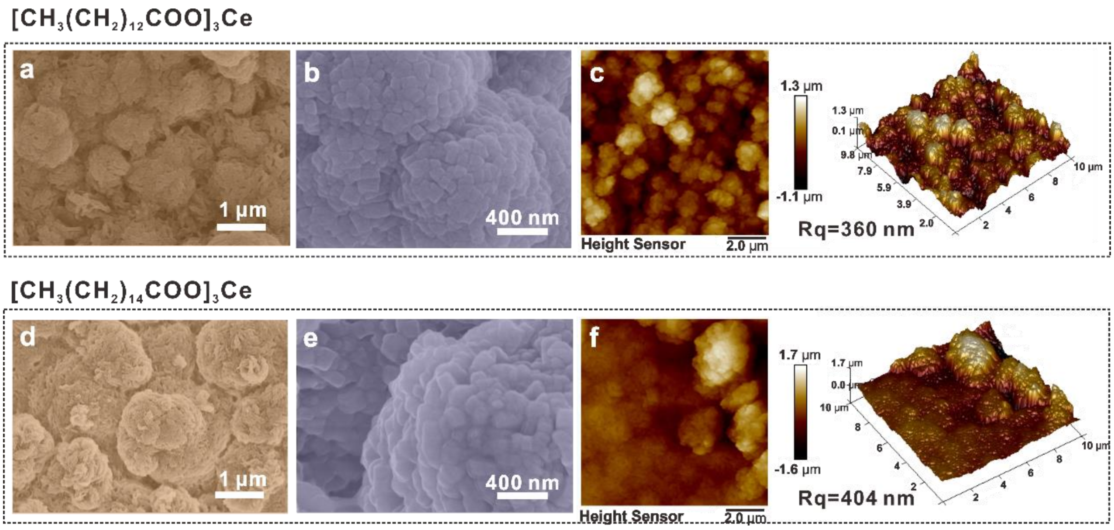

Supplementary Figure 9. **The morphology of super-hydrophobicity surfaces with different surface energies observed through SEM and AFM.** **a~c** $[CH_3(CH_2)_{12}COO]_3Ce$. **d~f** $[CH_3(CH_2)_{14}COO]_3Ce$.

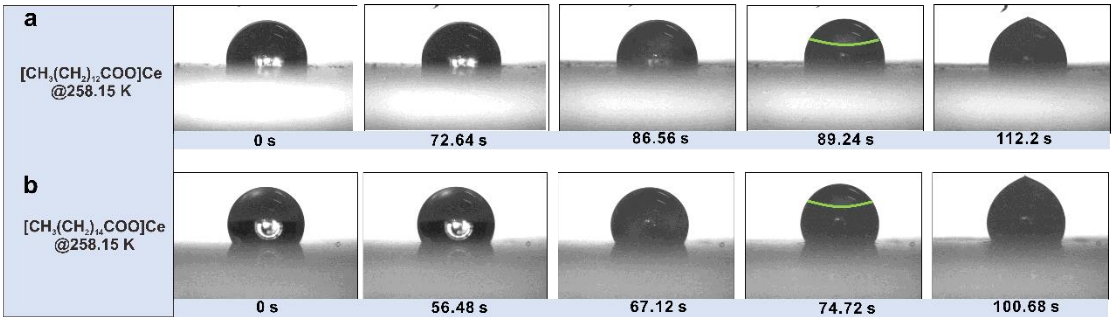

Supplementary Figure 10**. Icing delay time process on different sample surfaces**. **a** $[CH_3(CH_2)_{12}COO]_3Ce$@ 258.15 K. **b** $[CH_3(CH_2)_{14}COO]_3Ce$@ 258.15 K.

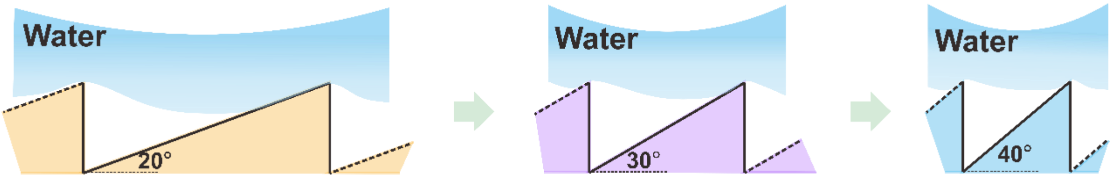

Supplementary Figure 11. **Diagram of wetting behavior of superhydrophobic surfaces with different microstructures.**

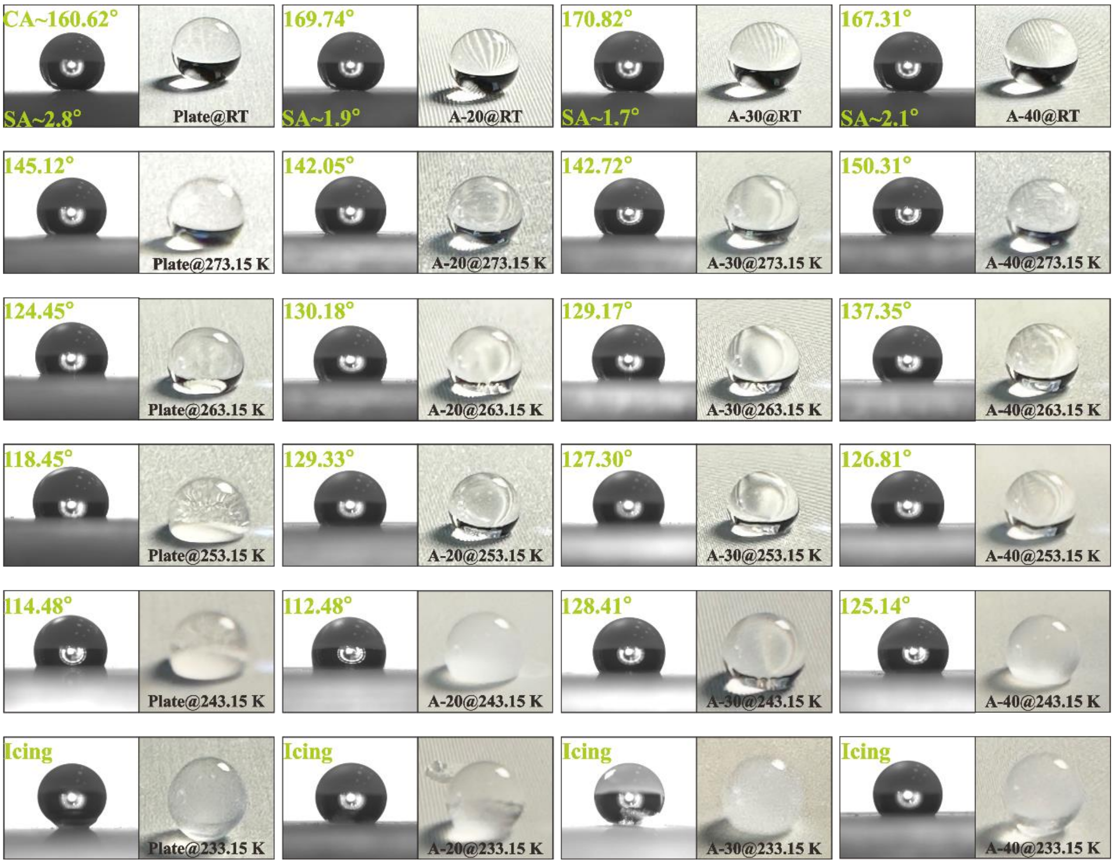

Supplementary Figure 12. **The wettability and mobility of the various superhydrophobic surface with nanostructure under different temperature conditions.**

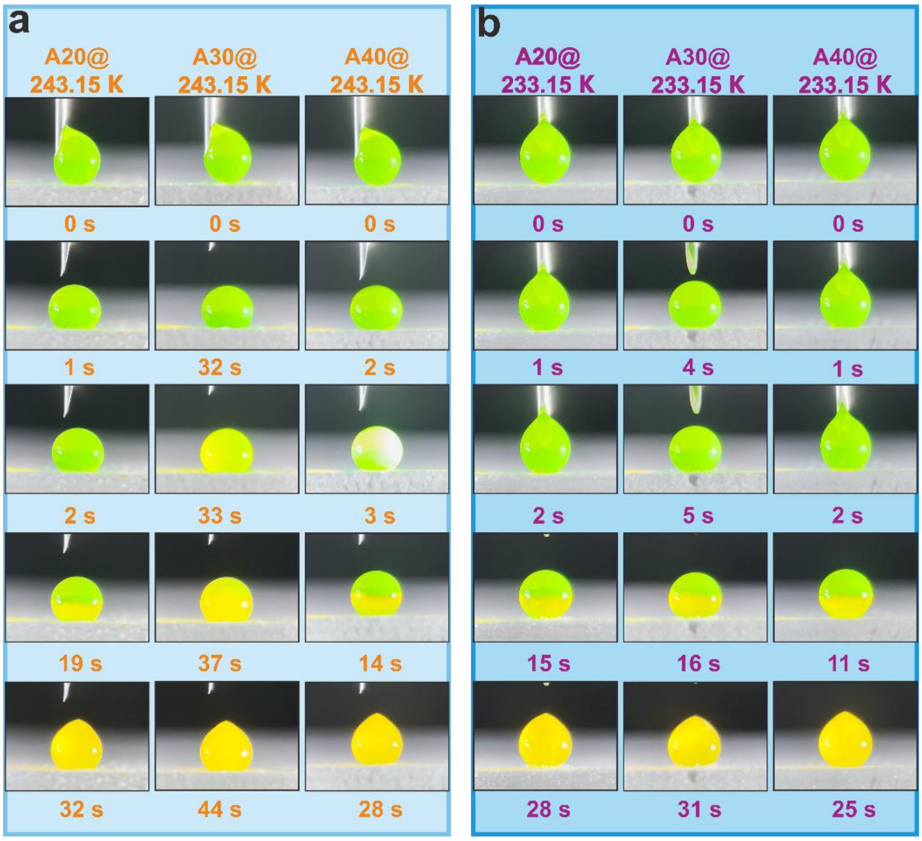

Supplementary Figure 13. **Droplet frozen on the Superhydrophobic surface at 243.15 K and 233.15 K.**

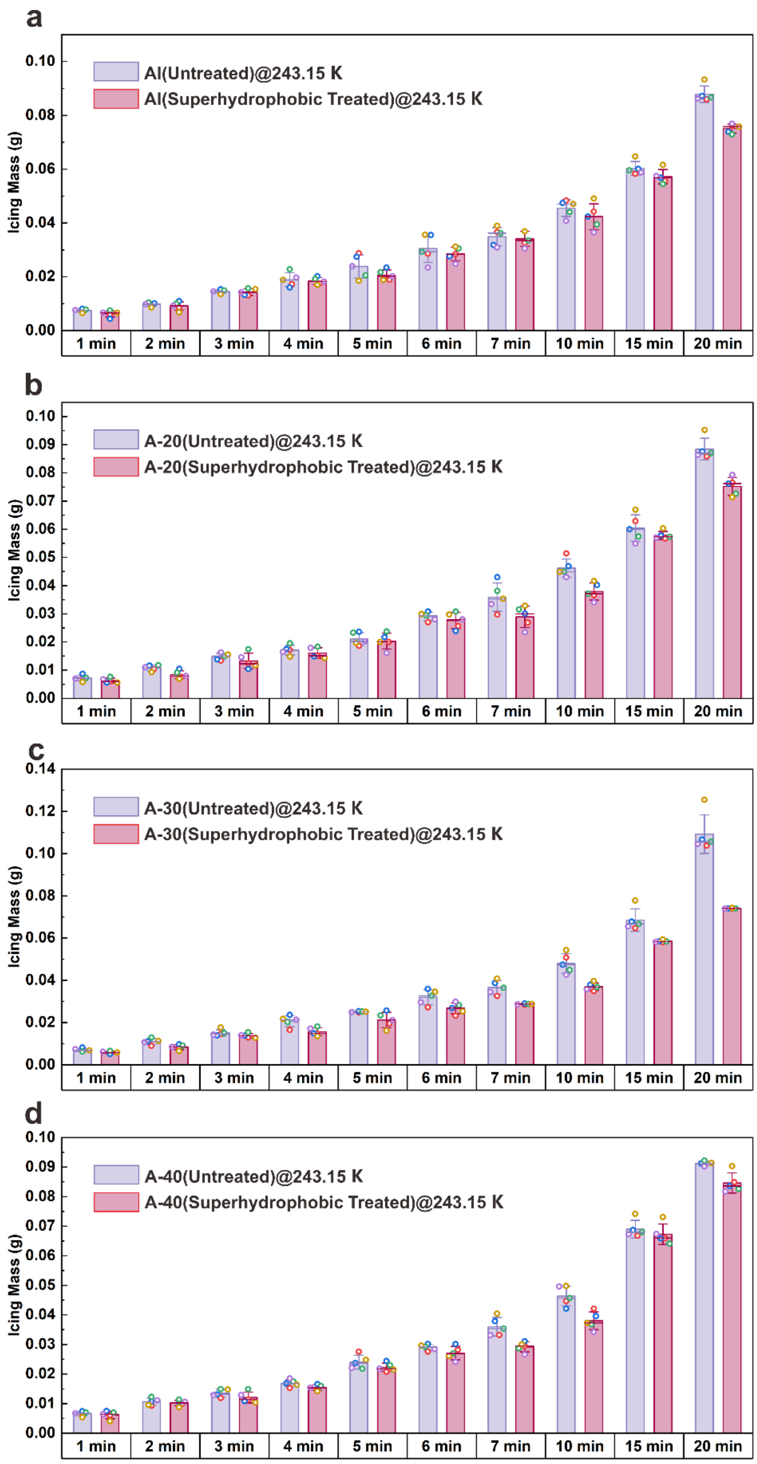

Supplementary Figure 14. **Ice accumulation on different samples at 243.15 K**. **a** Al. **b** A-20 sample. **c** A-30 sample. **d** A-40 sample. Error bars represent standard deviation. All the samples are tautologically measured for 5 times and averaged to mitigate potential error. The small circles in the graphs correspond to raw data.

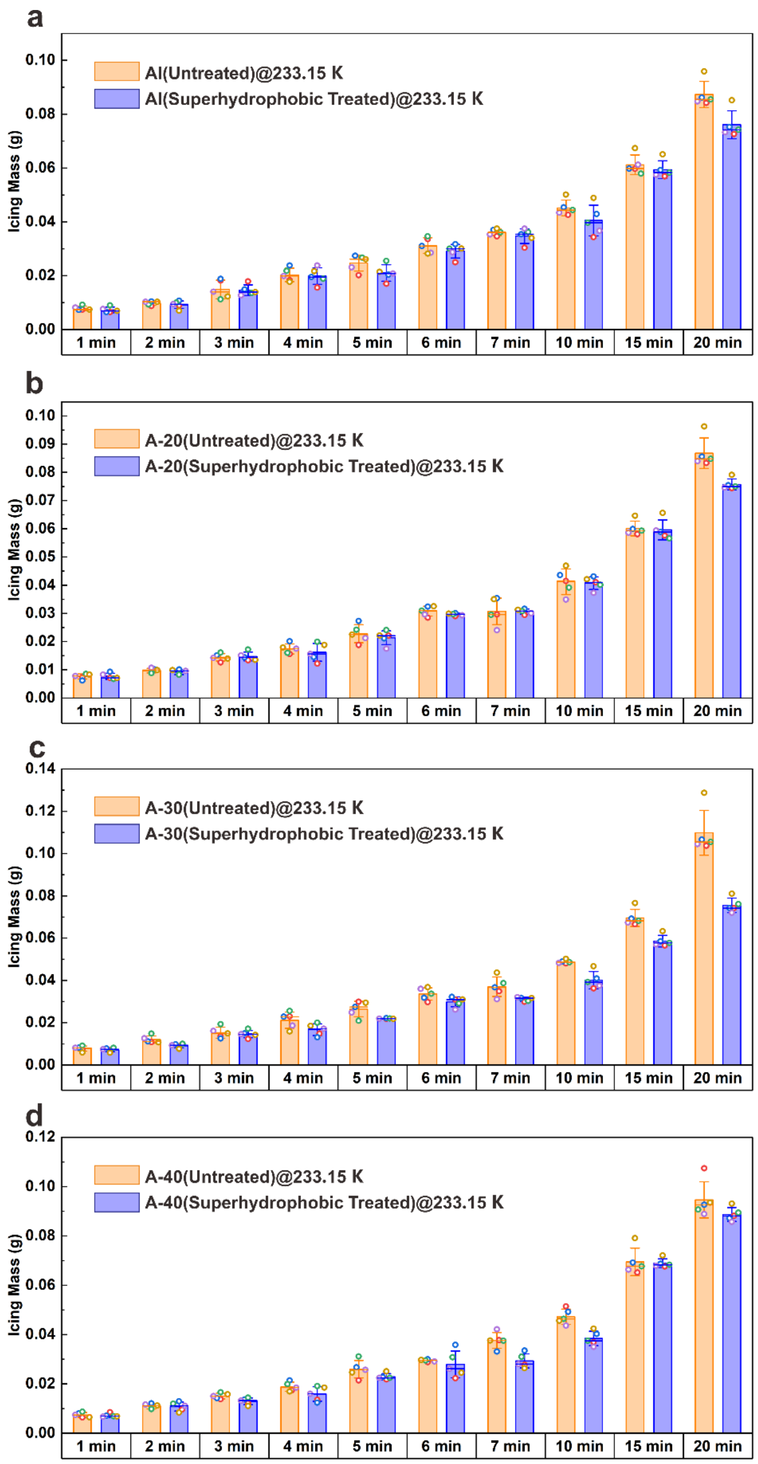

Supplementary Figure 15. **Ice accumulation on different samples at 233.15 K**. **a** Al. **b** A-20 sample. **c** A-30 sample. **d** A-40 sample. Error bars represent standard deviation. All the samples are tautologically measured for 5 times and averaged to mitigate potential error. The small circles in the graphs correspond to raw data.

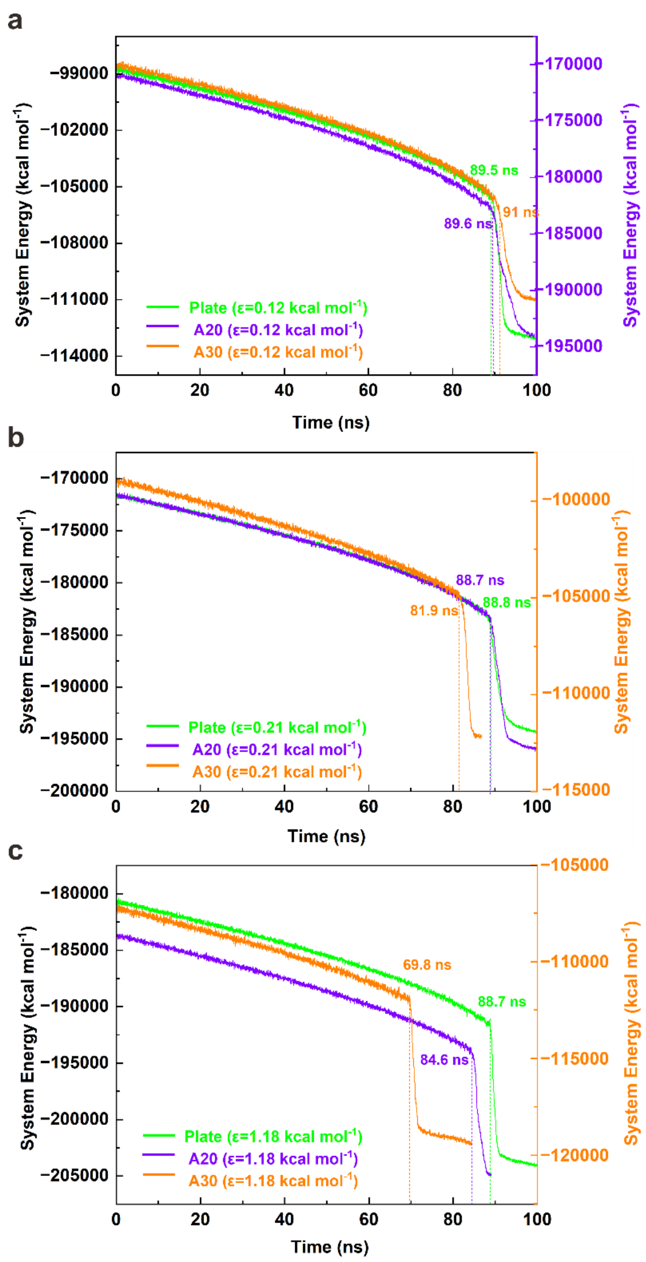

Supplementary Figure 16. **The energy curve of icing process. a** Icing process on different model with the surface interaction energy of 0.12 kcal mol^{-1}. **b** Icing process on different model with the surface interaction energy of 0.21 kcal mol^{-1}. **c** Icing process on different model with the surface interaction energy of 1.18 kcal mol^{-1}.

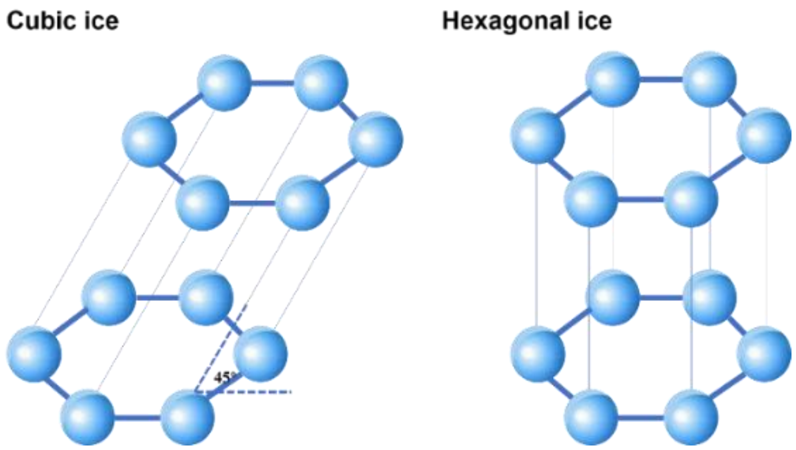

Supplementary Figure 17. **Structure diagram of cubic ice and hexagonal ice.**

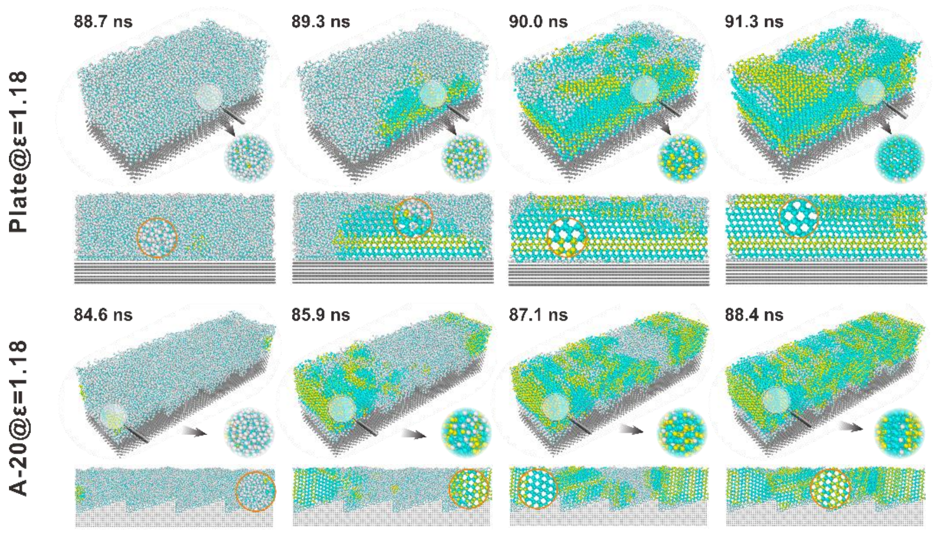

Supplementary Figure 18. **Icing process on plate@ε=1.18 kcal mol^{-1} and A-20@ε=0.12 kcal mol^{-1}.** The arrangement of typical water molecules along the vertical view and front view is amplified within circular regions. The evolution of the initial nucleation area (marked by magnifying glass) in the general view is enlarged in the circle regions on the right, as indicated by the black arrow.

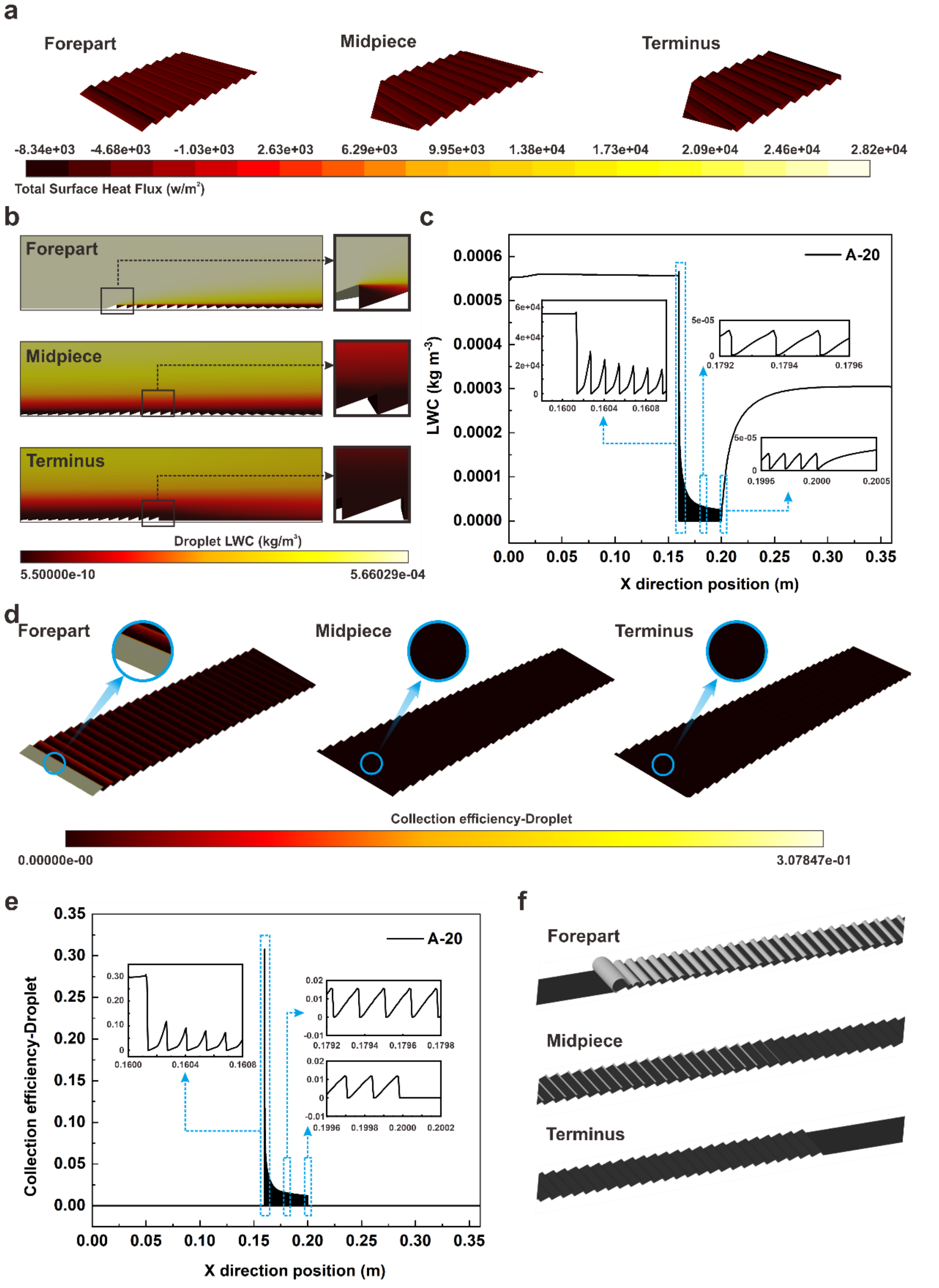

Supplementary Figure 19. **The CFD simulation of microdroplets movement under flow condition**. **a** Heat flux of A-20 sample. **b** LWC image of A-20 sample. The distribution of LWC in the near-wall regions of the microstructure is magnified and shown in the box on the right. **c** Corresponding LWC curves

of plate and A-20 sample. The LWC curves at specific locations (forepart, midpiece, and terminus of the structure region) are marked by dashed boxes and amplified respectively. **d** Droplet collection coefficient of A-20 sample. The droplet collection coefficient on the microstructure is amplified and displayed in the circular regions. **e** Droplet collection coefficient curves of A-20 sample. The curves of droplet collection coefficient at specific locations (forepart, midpiece, and terminus of the structure region) are marked by dashed boxes and amplified respectively. **f** The ice morphologies at the corresponding positions (forepart, midpiece, and terminus of the structure region).

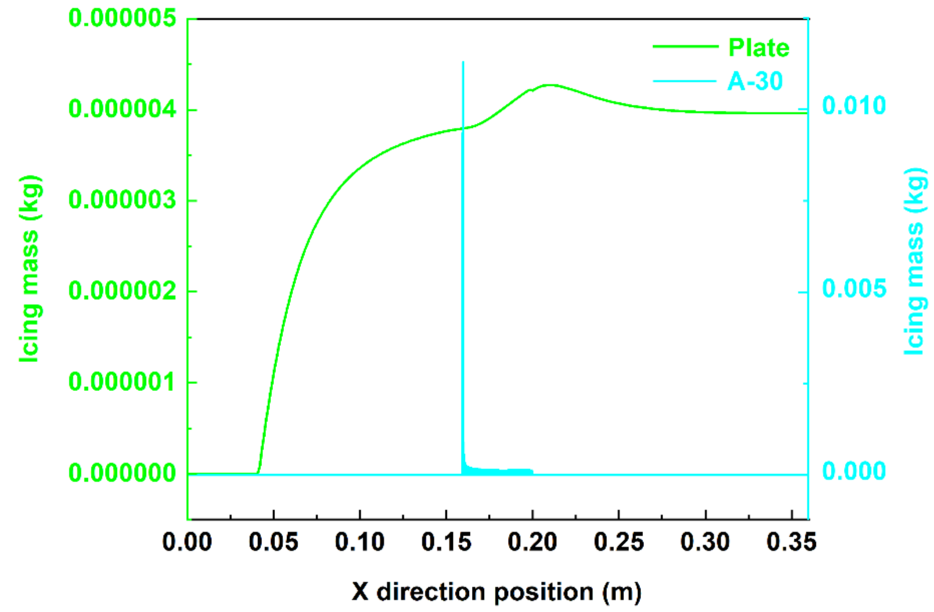

Supplementary Figure 20. **The icing mass curves of plate and A-30 sample.**

Icing behavior exploration of microdroplet in high-speed icing wind tunnel.

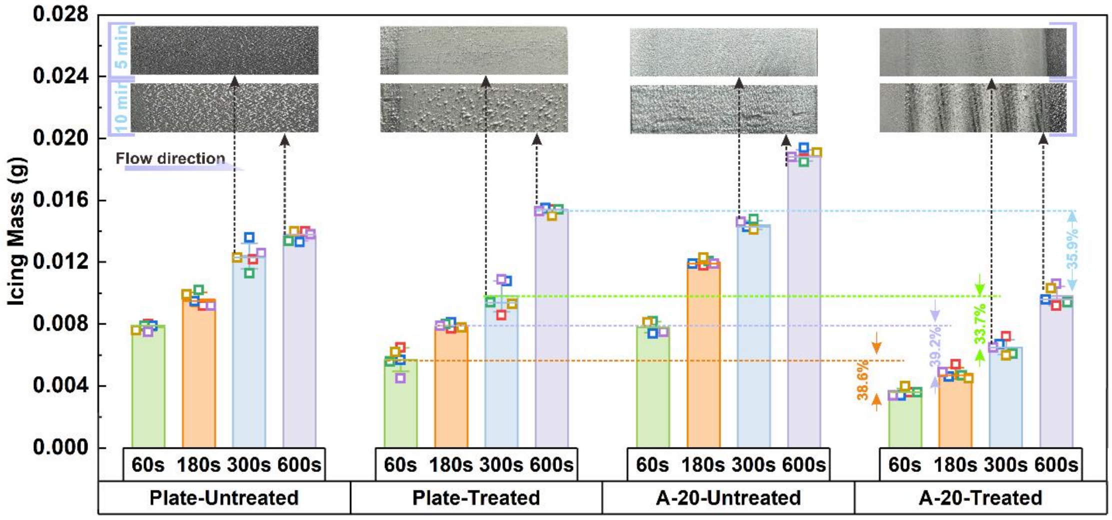

Supplementary Figure 21. **Ice accumulation quality and morphology of A-20 and plate samples in an ice wind tunnel.** The icing morphologies on the surfaces with the icing time of 300 s and 600 s are demonstrated in the upper part of the graphic respectively. The difference in ice accumulation between superhydrophobic A-20 sample and plate under various icing time is represented by corresponding colored numbers (60 s~49.1%, 180 s~48.1%, 300 s~40.8% and 600 s~43.8%).Error bars represent standard deviation. All the samples are tautologically measured for 5 times and averaged to mitigate potential error. The small square boxes in the graphs correspond to raw data.

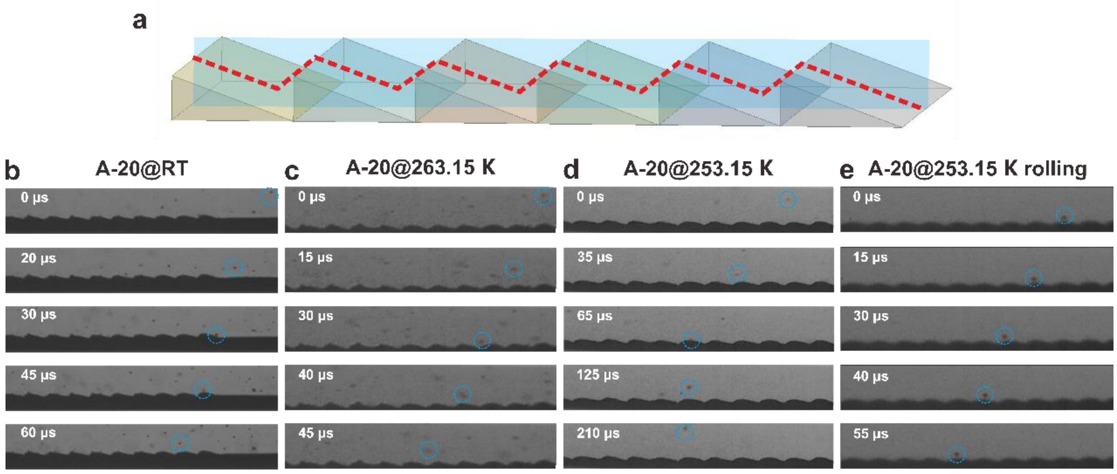

Supplementary Figure 22**. Microdroplet movement on the A-20 surface under different temperature**. **a** Observation angles of observation. **b** Microdroplet movement at room temperature. The moving microdroplets are marked by blue dotted circles. **c** Microdroplet movement at 263.15 K. **d** Microdroplet bouncing behavior at 253.15 K. **e** Microdroplet rolling behavior at 253.15 K.

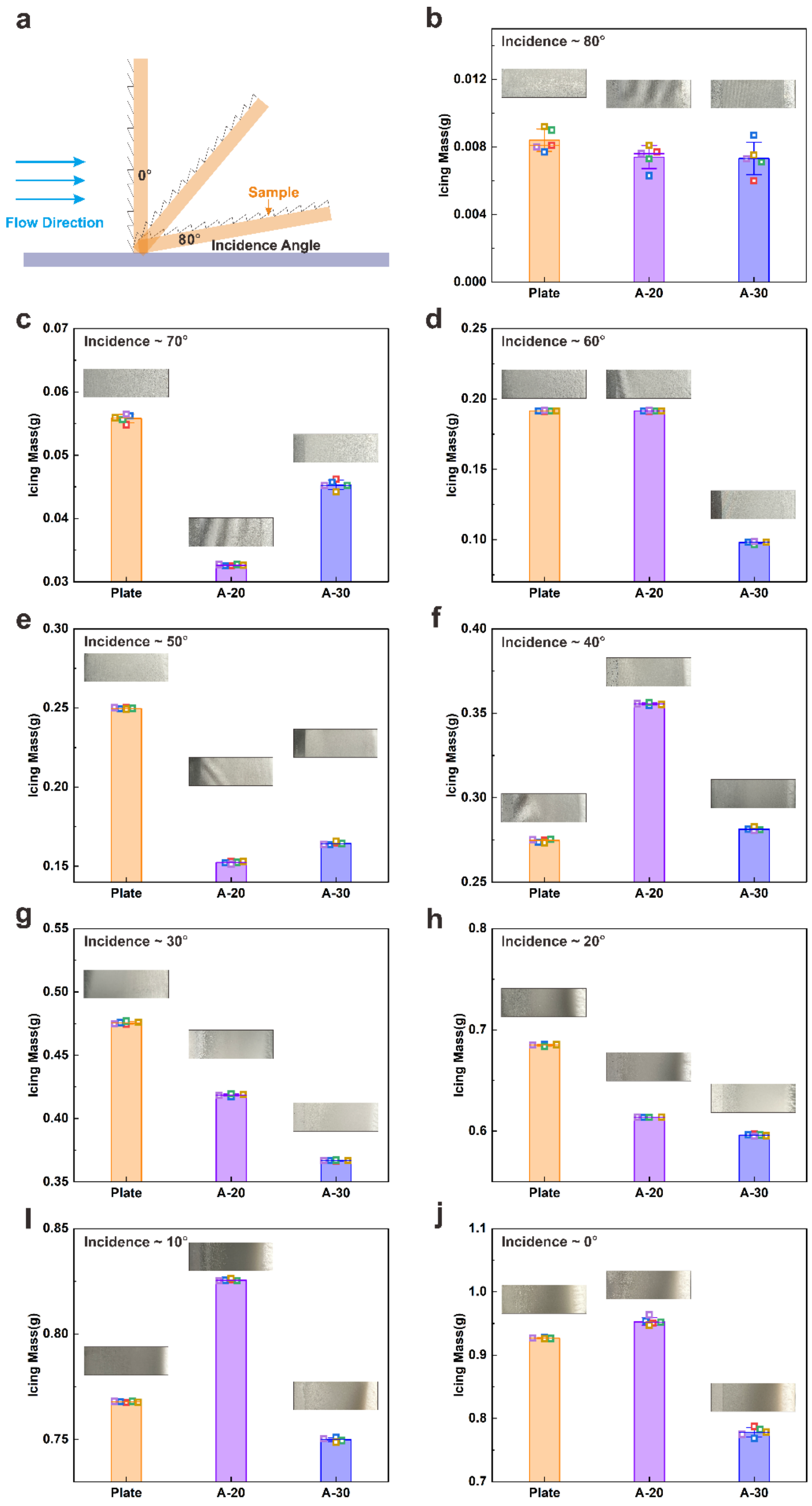

Supplementary Figure 23. **Anti-icing test with various incident angle**. **a** Schematic diagram of the test process. **b** Icing behavior of typical samples at

an incident angle of 80°. **c** Icing behavior of typical samples at an incident angle of 70°. **d** Icing behavior of typical samples at an incident angle of 60°. **e** Icing behavior of typical samples at an incident angle of 50°. **f** Icing behavior of typical samples at an incident angle of 40°. **g** Icing behavior of typical samples at an incident angle of 30°. **h** Icing behavior of typical samples at an incident angle of 20°. **i** Icing behavior of typical samples at an incident angle of 10°. **j** Icing behavior of typical samples at an incident angle of 0°. The icing Morphologies are inserted above the icing mass data for the corresponding sample. All the samples are tautologically measured for 5 times and averaged to mitigate potential error. Error bars represent standard deviation. All the small square boxes in the graphs correspond to raw data.

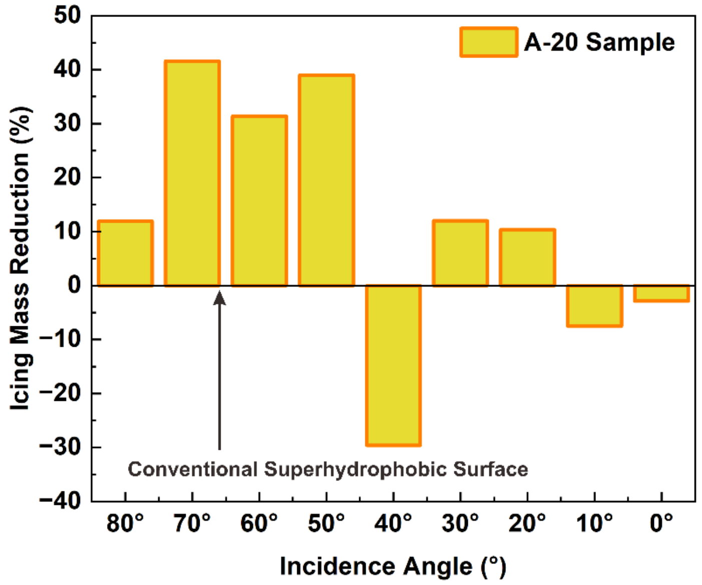

Supplementary Figure 24. **Icing mass reduction of A-20 sample.**

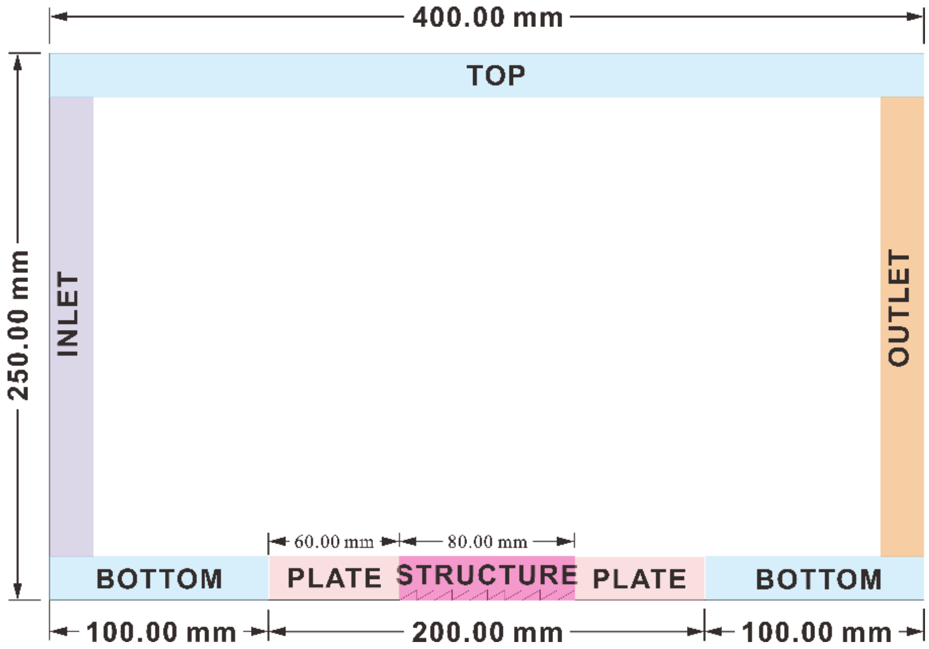

Supplementary Figure 25. **Numerical simulation domain of microstructure surface.**

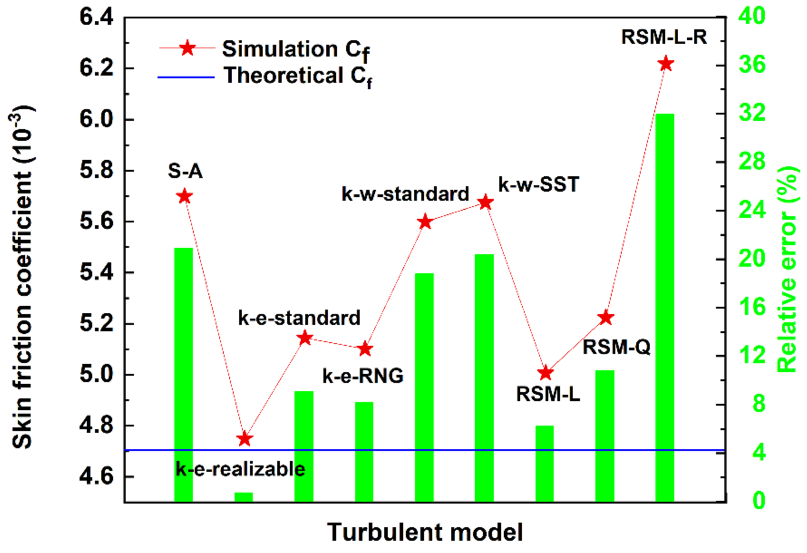

Supplementary Figure 26. **The comparison between the theoretical values and the calculated values under different turbulence models.**

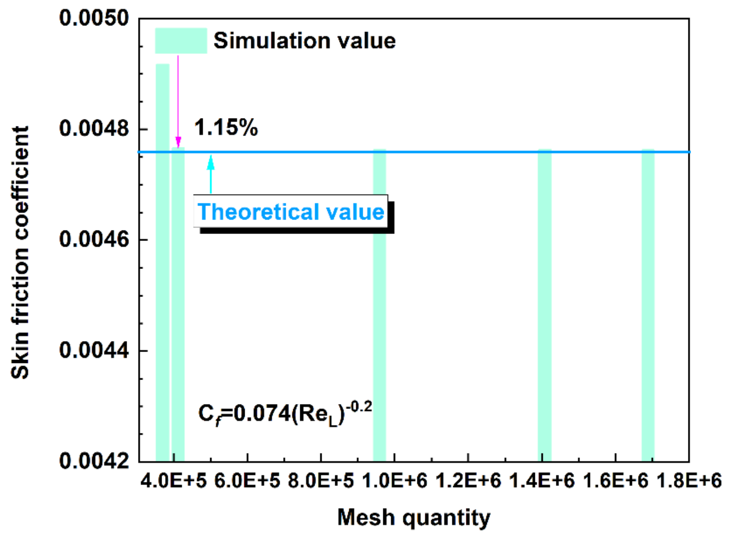

Supplementary Figure 27. **Validation of the grid independence.**

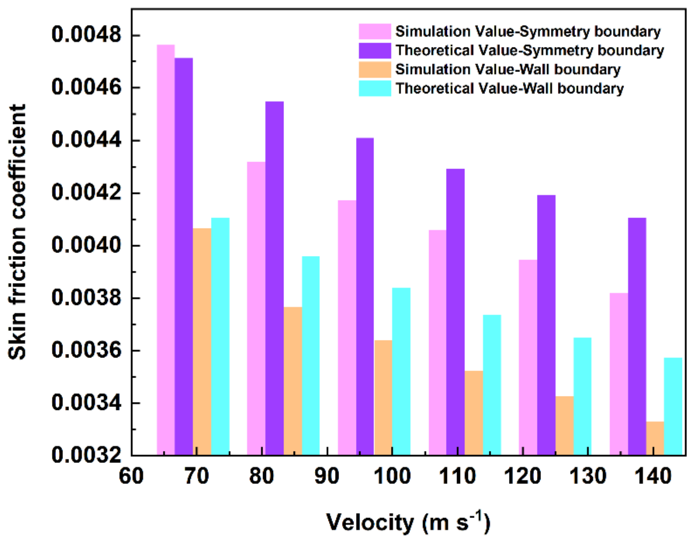

Supplementary Figure 28. **Comparison between calculated values and theoretical values.**

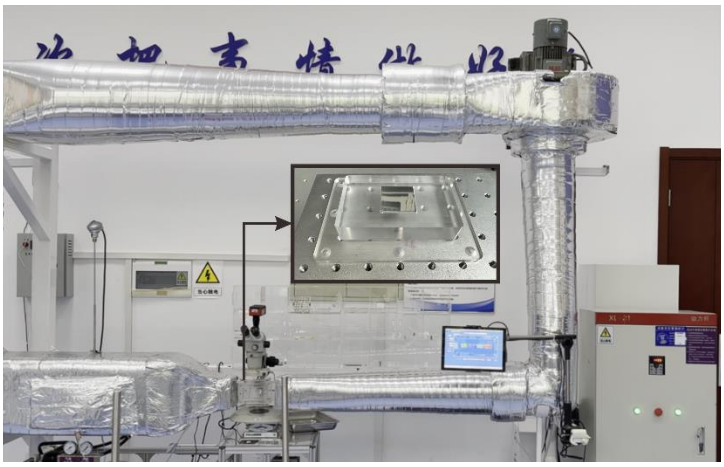

Supplementary Figure 29. **The test wind tunnel used in our work.**

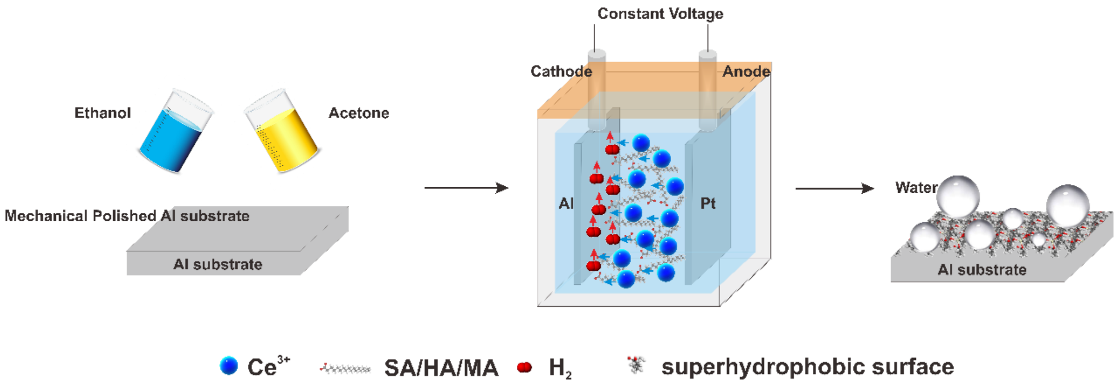

Supplementary Figure 30. **The preparation of superhydrophobic surface.**

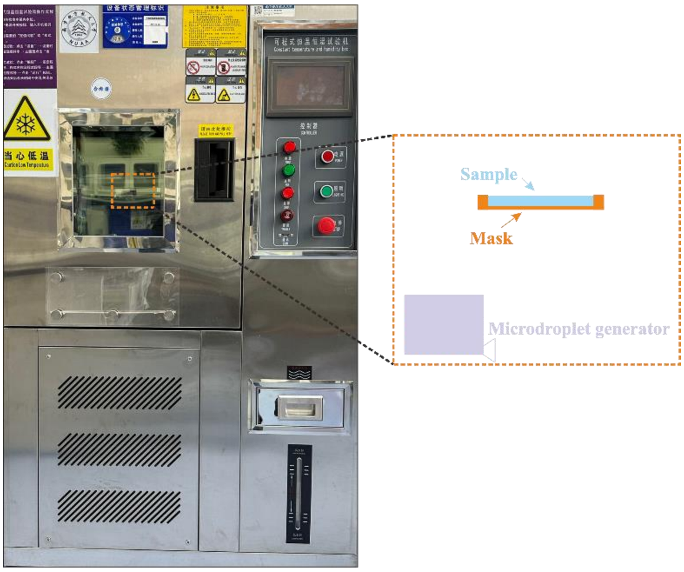

Supplementary Figure 31. **Self-made icing environment system.**

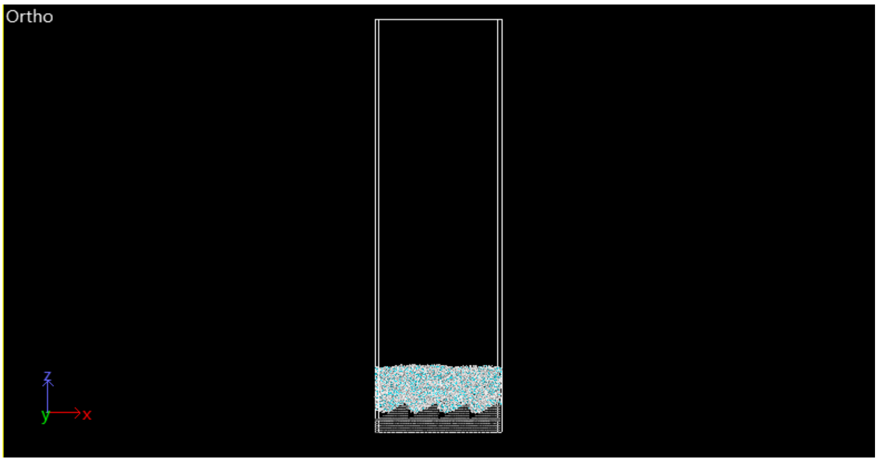

Supplementary Figure 32. **The diagram of model setup.**

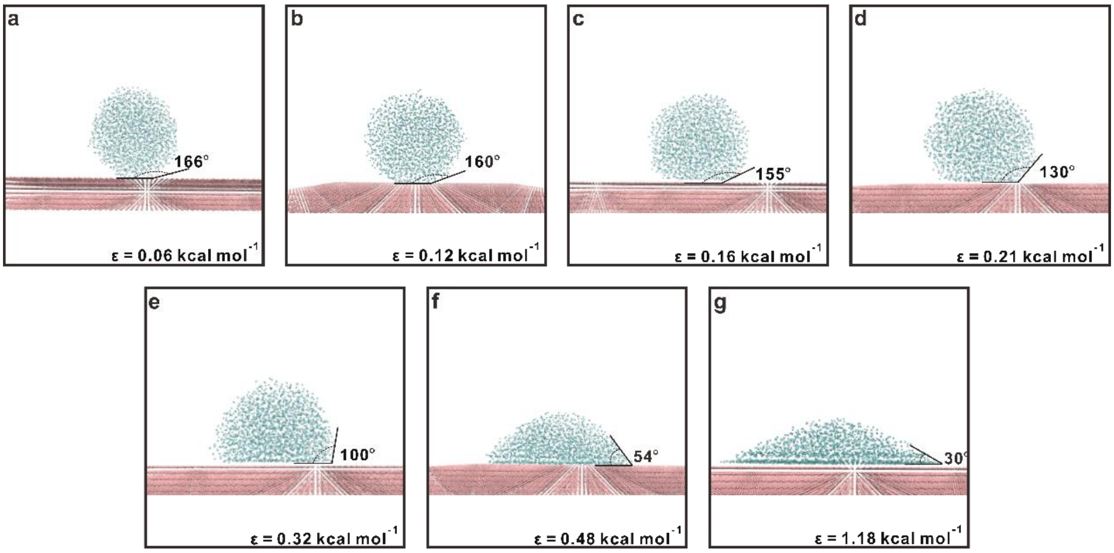

Supplementary Figure 33. **Contact angles of plate surface under different interaction energy**. **a** ε=0.06 kcal mol^{-1}. **b** ε=0.12 kcal mol^{-1}. **c** ε=0.16 kcal mol^{-1}. **d** ε=0.21 kcal mol^{-1}. **e** ε=0.32 kcal mol^{-1}. **f** ε=0.48 kcal mol^{-1}. **g** ε=1.18 kcal mol^{-1}.

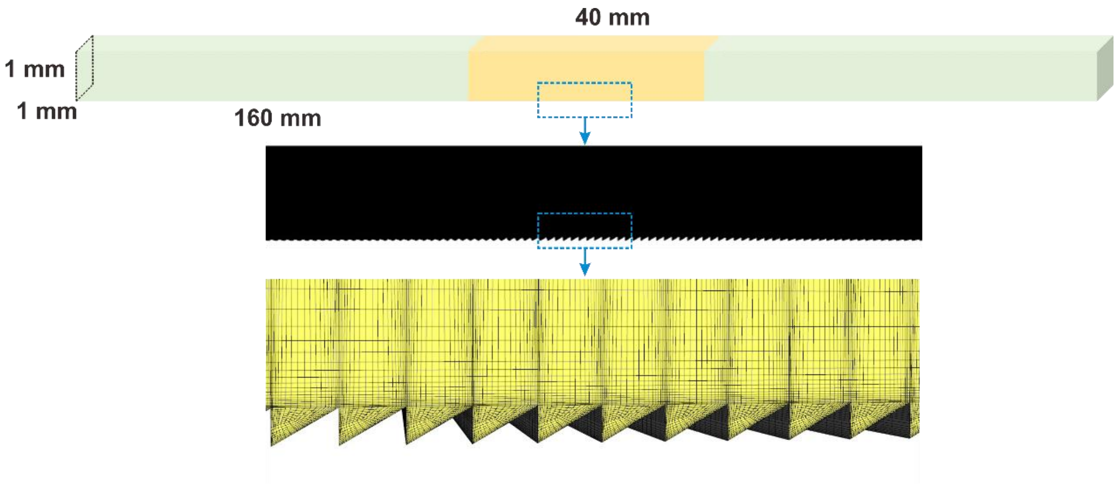

Supplementary Figure 34. **Numerical simulation domain of icing behavior under low temperature.** The region marked by the blue dashed line at the top of the figure is highlighted and enlarged to show the detailed grid structure of the array microstructure.

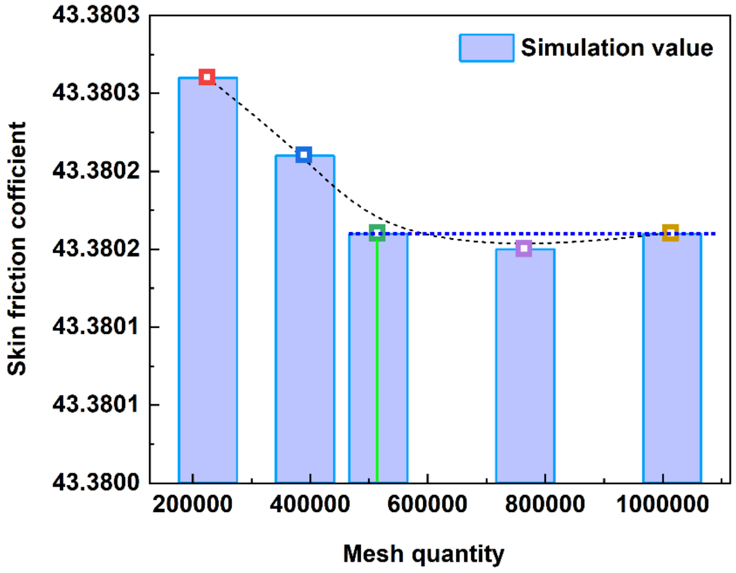

Supplementary Figure 35. **Validation of the grid independence.**

4. Supplementary Tables

Supplementary **Table 1**. **Summary of simulation parameters of the substrate and the water-substrate interaction parameters.**

**Sample**	**Surface**	**a_{fcc} (Å)**	**ε_{ws} (kcal mol^{-1})**	**σ_{ws} (Å)**	**Gravity ((kcal mol^{-1}) Angstrom^{-1})**
1	FCC(100)	2.866	1.18	2.488	6.24×10^{-4}
2	FCC(100)	2.866	0.21	2.488	6.24×10^{-4}
3	FCC(100)	2.866	0.12	2.488	6.24×10^{-4}

5. Supplementary References

[1] Song X W, Zhang M X, Lin P Z. Skin Friction Reduction Characteristics of Nonsmooth Surfaces Inspired by the Shapes of Barchan Dunes. *Mathematical Problems in Engineering* (2017).

[2] Matsumoto M, Saito S, Ohmine I. Molecular dynamics simulation of the ice nucleation and growth process leading to water freezing. *Nature* 416, 409-413 (2002).

[3] Kramer, B. et al. Homogeneous nucleation rates of supercooled water measured in single levitated microdroplets. *J. Chem. Phys.* 111, 6521–6527 (1999).

[4] Bartell, L. S. Nucleation rates in freezing and solid-state transitions. *J. Phys. Chem*. 99, 1080–1087 (1995).

[5] Abraham, F. F. Homogeneous Nucleation Theory. Academic. New York. (1974).

[6] Xu Y, Shen Y, Tao J, et al. Selective nucleation of ice crystals depending on the inclination angle of nanostructures. *Phys. Chem. Chem. Phys*. 22, 1168-1173 (2020).

[7] Sauermann G, Andrade Jr J S, Maia L P, et al. Wind velocity and sand transport on a barchan dune. *Geomorphology* 54, 245-255 (2003).

[8] Bishop M A. Point pattern analysis of north polar crescentic dunes, Mars: A geography of dune self-organization. *Icarus* 191, 151-157 (2007).

[9] Durán O, Claudin P, Andreotti B. On aeolian transport: Grain-scale

interactions, dynamical mechanisms and scaling laws. *Aeolian Res*. 3, 243-270 (2011).

[10] Douady S. Equilibrium versus disequilibrium of barchan dunes. *Geomorphology* 125, 558-568 (2011).

[11] Finkel H J. The barchans of southern Peru. *The journal of geology* 67, 614-647 (1959).

[12] Sauermann G, Rognon P, Poliakov A, et al. The shape of the barchan dunes of Southern Morocco. *Geomorphology* 36, 47-62 (2000).

[13] Jiang Jiawei, Shen Yizhou, Tao Jie, Jia Zhenfeng, Xie Xinyu, Zeng Chaojiao. Drag reduction characteristics of microstructure inspired by the shapes of barchan dunes under high speed flow condition. *Journal of Renewable Materials* 10, 781-797 (2021).

[14] Faheem A, Ranzi G, Fiorito F, et al. A numerical study of turbulent mixed convection in a smooth horizontal pipe. *J. Heat Transfer*. 138 (2016).

[15] Shih T H, Liou W W, Shabbir A, et al. A new k-epsilon eddy viscosity model for high Reynolds number turbulent flows: Model development and validation. (1994).

[16] Li M, Khan T S, Al-Hajri E, et al. Single phase heat transfer and pressure drop analysis of a dimpled enhanced tube. *Appl. Therm. Eng*. 101, 38-46 (2016).

[17] Shih, T.H., Liou, W.W., Shabbir, A., Yang, Z., Zhu, J. A new k-epsilon eddy viscosity model for high Reynolds number turbulent flows. *Comput Fluids*.

24, 227-238 (1995).

[18] Launder, B.E., Spalding, D.B. The numerical computation of turbulent flows. Numerical prediction of flow, heat transfer, turbulence and combustion. *Pergamon* 96-116 (1983).

[19] Bi Y, Cao B, Li T. Enhanced heterogeneous ice nucleation by special surface geometry. *Nat. Commun*. 8, 15372 (2017).

[20] Haji-Akbari A, DeFever R S, Sarupria S, et al. Suppression of sub-surface freezing in free-standing thin films of a coarse-grained model of water. *Phys. Chem. Chem. Phys*. 16, 25916-25927 (2014).

[21] M. Fitzner, G. C. Sosso, F. Pietrucci, S. Pipolo, A. Michaelides. Pre-critical fluctuations and what they disclose about heterogeneous crystal nucleation. *Nat. Commun*. 8, 2257 (2017).

[22] Lupi L, Hudait A, Molinero V. Heterogeneous Nucleation of Ice on Carbon Surfaces. *J. Am. Chem. Soc*. 136, 3156-3164 (2014).

[23] Li N, Jiang J, Yang M Y, et al. Anti-icing mechanism of combined active ethanol spraying and passive surface wettability. *Appl. Therm. Eng*. 220, 119805 (2023).

[24] Maras E, Trushin O, Stukowski A, et al. Global transition path search for dislocation formation in Ge on Si (001). *Comput. Phys. Commun*. 205, 13-21 (2016).

[25] Aupoix B. Roughness Corrections for the k–ω Shear Stress Transport Model: Status and Proposals. *Mathematical Notes* 20, 240 (2015).
