# Supplementary material for: An energy-free strategy to elevate anti-icing performance of superhydrophobic materials through interfacial airflow manipulation
Source: Nat Commun. 2024 Jan 26;15:777. doi: 10.1038/s41467-024-45078-5 (PMC10817900; doi:10.1038/s41467-024-45078-5)
Supplement: Supplementary file 3 — Description of Additional Supplementary Files [file 41467_2024_45078_MOESM3_ESM.pdf]

**Description of Additional Supplementary Files**

**File Name:** Supplementary Movie 1
**Description:** Icing behavior on the plate with a ε of 0.12.

**File Name:** Supplementary Movie 2
**Description:** 2 Icing behavior on the A-30 model with a ε of 0.12.

**File Name:** Supplementary Movie 3
**Description:** Icing behavior on the A-30 model with a ε of 1.18.

**File Name:** Supplementary Movie 4
**Description:** Microdroplets movement on the superhydrophobic plate.

**File Name:** Supplementary Movie 5
**Description:** Microdroplets movement on the superhydrophobic A30 sample.
